# Cesarean delivery rates, hospital readiness and quality of clinical management in Ethiopia: national results from two cross-sectional emergency obstetric and newborn care assessments

**DOI:** 10.1186/s12884-021-04008-9

**Published:** 2021-08-19

**Authors:** Misrak Getnet Beyene, Theodros Getachew Zemedu, Azmach Hadush Gebregiorgis, Ana Lorena Ruano, Patricia E. Bailey

**Affiliations:** 1 Ethiopian Public Health Institute, Addis Ababa, Ethiopia; 2 World Health Organization, Lusaka, Zambia; 3 Centre for International Health, Department of Global Public Health and Primary Care, University of Bergen, Bergen, Norway; 4 Consultant with Averting Maternal Death and Disability Program, Mailman School of Public Health, Columbia University, New York, USA

**Keywords:** Cesarean delivery, Cesarean-section, Cesarean rates, Readiness, Robson classification, Public/private managing authority, Quality of care

## Abstract

**Background:**

Cesarean delivery (CD) rates have reached epidemic levels in many high and middle income countries while increasingly, low income countries are challenged both by high urban CD rates and high unmet need in rural areas. The managing authority of health care institutions often plays a role in these disparities. This paper shows changes between 2008 and 2016 in Ethiopian CD rates, readiness of hospitals to provide CD and quality of clinical care, while highlighting the role of hospital management authority.

**Methods:**

This secondary data analysis draws from two national cross-sectional studies to assess emergency obstetric and newborn care. The sample includes 111 hospitals in 2008 and 316 hospitals in 2016, and 275 women whose CD chart was reviewed in 2008 and 568 in 2016. Descriptive statistics are used to describe our primary outcome measures: population- and institutional-based CD rates; hospital readiness to perform CD; quality of clinical management, including the relative size of Robson classification groups.

**Results:**

The national population CD rate increased from 2008 to 2016 (< 1 to 2.7%) as did all regional rates. Rates in 2016 ranged from 24% in urban settings to less than 1% in several rural regions. The institutional rate was 54% in private for-profit hospitals in 2016, up from 46% in 2008. Hospital readiness to perform CDs increased in public and private for-profit hospitals. Only half of the women whose charts were reviewed received uterotonics after delivery of the baby, but use of prophylactic antibiotics was high. Partograph use increased from 9 to 42% in public hospitals, but was negligible or declined elsewhere. In 2016, 40% of chart reviews from public hospitals were among low-risk nulliparous women (Robson groups 1&2).

**Conclusions:**

Between 2008 and 2016, government increased the availability of CD services, improved public hospital readiness and some aspects of clinical quality. Strategies tailored to further reduce the high unmet need for CD and what appears to be an increasing number of unnecessary cesareans are discussed. Adherence to best practices and universal coverage of water and electricity will improve the quality of hospital services while the use of the Robson classification system may serve as a useful quality improvement tool.

**Supplementary Information:**

The online version contains supplementary material available at 10.1186/s12884-021-04008-9.

## Background

Increasingly, low and middle income countries face the two extremes of unmet need for and unnecessary cesareans, or “too little too late and too much too soon” [1]. The World Health Organization (WHO) began producing global guidance on this topic as early as 1985 [2]. In 2015, the WHO provided evidence that rates greater than 10% were not associated with a reduction in maternal and newborn mortality. At the same time, the WHO cautioned against pursuing specific targets and stressed that all women who need a CD should receive one [3, 4]. Women who have a cesarean undergo perioperative risks such as blood loss, anesthetic accidents, wound infection and iatrogenic fistula, and put future pregnancies at risk due to uterine scaring [5–7]. But where cesarean deliveries are too few, we see excessive short- and long-term maternal morbidity and maternal and newborn mortality that could be mitigated by timely surgical intervention.

Eastern and southern Africa has marginally increased its CD rate from 4.6 to 6.2% between 2000 and 2015 compared to most of Asia and Eastern Europe where rates more than doubled during this period, and in 2015, ranged from 18 to 29% [8]. Recent global interest and regional studies on surgery have highlighted extensive unmet need for surgery. They also documented safety concerns when undertaken too late or in environments where deficits in surgical personnel and resources are common, such as oxygen, medical equipment and electricity [9, 10].

Ethiopia is a country with high maternal and newborn mortality, high prevalence of fistula and historically low cesarean and institutional delivery rates. According to its 2016 Demographic and Health Survey, the CD rate was 2% based on births during the 5 years prior to the survey [11]. Earlier trend data from the capital indicated rising cesarean rates that were positively correlated with income and education, as in many other country settings [8, 12, 13]. The current Health Sector Transformation Plan set a population-based cesarean target of 8% for 2020 as a step towards addressing the unmet need for life-saving surgery [14].

This paper aims to update an earlier paper written by Fesseha and colleagues that described a national review of cesarean delivery in Ethiopia in 2008 [15]. The current paper compares 2008 with the situation in 2016 and describes changes in national and regional population CD rates; institutional CD rates; the readiness of hospitals to perform obstetric surgery; and the quality of clinical management of cesarean delivery, while highlighting differences across hospital management.

## Methods

### Study design, setting and data collection

This secondary data analysis draws on two national cross-sectional surveys or assessments of emergency obstetric and newborn care (EmONC) from 2008 and 2016 [16, 17]. Each was a census of public and private health facilities that met government criteria to provide childbirth services.

The data collection instruments for 2008 were adapted from a set of standard modules previously used in many countries [18]. The 2016 assessment used the same core questionnaires administered in 2008 that subsequently underwent a global revision with local adaptation in 2016. The six modules relevant to this study were: M1 - basic health facility infrastructure; M2 - human resources; M3 - inventory of drugs, equipment and supplies; M4 - summary service statistics; M5 - performance of signal functions; and M8 – chart review for CDs. All facilities received modules 1–5; only facilities that performed CDs received M8. The latter was modified in 2016 to include information not in 2008 such as a woman’s characteristics needed to classify her into one of the 10 Robson groups, as well as type of anesthesia and professional cadre who performed the operation. See supplementary files 1, 2, 3, 4, 5 and 6 for the modules.

The first assessment was launched 1 October 2008 and completed by 15 January 2009; the 2016 assessment commenced in mid-May and was completed by mid-December 2016. In 2008, a private company conducted the assessment and prepared the databases [16] and in 2016, this responsibility shifted to the Ethiopian Public Health Institute. Details about the data collector training and survey execution can be found in the final reports and previously published papers [15–17, 19].

### Study population

This paper features three study populations or units of analysis: 1) aggregated hospital service data, 2) hospitals and 3) individual women who delivered by cesarean.

Aggregated service data consisted of the number of women who delivered in each facility by mode of delivery and covered 12 consecutive months prior to the assessment (July 2007–June 2008 and January–December 2015). These data were used to estimate population and institutional CD rates. The 12-month tally of the number of women who received a cesarean delivery in 2008 was 17, 145 and the total number who delivered in these same facilities during the same time period were 93,689. In 2016 those numbers increased to 78,916 cesarean deliveries among 343,492 institutional deliveries.

The second study population was the health facility. If the Ethiopian Food, Medicine and Health Control Authority approved a facility as a site to deliver routine and/or operative childbirth services, it was eligible for the assessment. Between 2008 and 2016 the Ministry of Health led a massive infrastructure expansion, adding about 3000 health centers and 200 hospitals [20, 21]. In 2008, 751 health facilities with childbirth services were visited, including 111 hospitals. In 2016, 3804 health facilities were visited, 316 of which were hospitals. This paper focuses exclusively on the hospitals.

Although we call both assessments a census, in 2008, 15 facilities were not visited because they did not appear on the master list of licensed facilities, 12 of which were in the capital of Addis Ababa. In 2016, 11 facilities were not visited due to civil unrest but few if any of these service sites were hospitals. Finally, two hospitals refused to participate in the 2016 assessment. Nevertheless, we consider our estimates to be based on national “populations” of health facilities and of women who gave birth in facilities rather than samples.

The third study population was defined by the women whose cesarean delivery was selected for review. In each hospital that had performed cesarean delivery, a small sample of women who delivered by cesarean had their charts reviewed. In 2008 data collectors identified three women per hospital and in 2016 only two cases per hospital were selected; the number was reduced due to the increase in the number of hospitals and the time required to complete chart reviews. The selection criteria were the same for each assessment: 1) cases occurred in the previous 12 months, and 2) they were the last women who had had a cesarean but were no longer under postoperative care, regardless of survival. Although the cases were systematically chosen, technically they were a convenience sample. In 2008, 275 women who underwent CD were reviewed and in 2016 that number was 568.

### Processes and comparisons

#### Variables, readiness definition and Robson classification

Our key stratifying variable across all analyses was hospital managing authority, defined by three categories of hospitals: public or government hospitals, private for-profit hospitals and private not-for-profit hospitals (managed by non-governmental organizations and/or religious missions).

For both population and institutional CD rates, the numerator was the sum of all CDs performed at each hospital. The denominator for the population rate was the number of expected births in each region, based on population figures from the Planning and Programming Department of the Federal Ministry of Health [20, 22]. The population figures were multiplied by the crude birth rate established by the 2016 Demographic and Health Survey and FMoH reports in 2008 [11, 22]. The use of expected births rather than known births is standard methodology for EmONC assessments since vital registration of all births is often incomplete [23]. The denominator for the institutional CD rate was the sum of all births at each hospital that reported having provided cesarean services in module 4.

Modules 1, 2, 3 and 5 provided information to assess hospital readiness to perform CD. To assess readiness, we created a binary summary score (yes or no), based on 1) the availability of at least one health professional able to perform the operation and another individual to provide anesthesia, plus 2) functional readiness items. The latter included EITHER an anesthesia machine + (halothane or ketamine) OR regional anesthesia (lignocaine/ lidocaine 4% or bupivacaine) AND an oxygen cylinder with manometer and flowmeter (low flow) tubes and connectors, an operating table and a functioning adjustable light. Although not included in the summary score of readiness requirements, interruptions in water and electricity in the operation theaters were assessed also.

Information on the quality of clinical management was drawn from module 8 and was measured by the use of a partograph, administration of prophylactic antibiotics and uterotonics, time interval from decision to incision, type of anesthesia and the clinician who performed the CD as well as maternal and newborn outcomes.

**Fig. 1:**
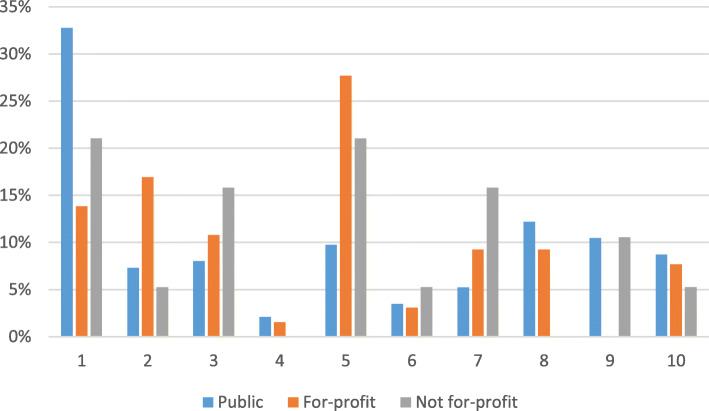
Relative size of the 10 Robson groups by managing authority (*n* = 371), Ethiopia 2008 and 2016. Group 1: parity 0, no previous CD, singleton, cephalic, ≥ 37 weeks, spontaneous labor. Group 2: parity 0, no previous CD, singleton, cephalic, ≥ 37 weeks, induced or CD before labor. Group 3: parity ≥ 1, no previous CD, singleton, cephalic, ≥ 37 weeks, spontaneous labor. Group 4: parity ≥ 1, no previous CD, singleton, cephalic, ≥ 37 weeks, induced or CD before labor. Group 5: parity ≥ 1, previous CD, singleton, cephalic, ≥ 37 weeks, any onset of labor. Group 6: parity 0, no previous CD, singleton, breech, any gestational age, any onset of labor. Group 7: parity ≥ 1, yes or no previous CD, singleton, breech, any gestational age, any onset of labor. Group 8: any parity, yes or no previous CD, multiple fetuses, any fetal lie, any gestational age, any onset of labor. Group 9: any parity, yes or no previous CD, singleton, transverse or oblique lie, any gestational age, any onset of labor. Group 10: any parity, yes or no previous CS, singleton, cephalic, < 37 weeks, any onset of labor

A final analysis of clinical management was based on the Robson 10-group classification scheme, designed to determine institutional cesarean rates for clinically relevant and mutually exclusive groups [24]. The classification system depends on six characteristics of women that are easily captured: parity (nulliparous/multiparous), number of fetuses (singleton/multiple), onset of labor (spontaneous or induced/CD before labor started), previous CD (yes/no), fetal lie (cephalic/ transverse/breech) and gestational age (< 37 weeks/ ≥ 37 ). Because the 10 groups (see Fig. 1) tend to have different cesarean rates, the classification scheme is used to inform where changes in clinical management should be made. Our aim in using the Robson classification, however, was to determine the distribution of cases reviewed according to the 10 groups in order to show their relative size, if and how the group size varied across managing authority, and the extent to which the group sizes aligned with other studies.

#### Statistical analysis

To produce descriptive statistics (frequencies, percentages, means and medians) we used SPSS version 24. Missing data were usually reported. Since our data sources were censuses and not random samples, nor did they represent some theoretical population, we performed no statistical tests.

### Ethical considerations

This paper utilizes secondary data; permission to use the data was granted by the Ethiopian Public Health Institute and the Family Health Division at the Federal Ministry of Health.

## Results

In 2008, 87 of the 111 hospitals provided cesarean deliveries (66 public sector, 15 private for-profit and 6 private not-for-profit). In 2016, 253 out of 316 hospitals provided cesarean services (188, 52 and 13, respectively).

### Cesarean delivery rates

The national population cesarean rate in 2008 was 0.6 and 2.7% in 2016 (Table 1). The highly urbanized regions of Harari, Addis Ababa and Dire Dawa exhibited the highest rates in 2016: 17, 24, and 10%, respectively. Elsewhere, rates increased but remained very low.

**Table 1:** Absolute numbers of cesarean deliveries (CD), expected births and population-based CD rates, by region and year of assessment

Region	Public	Private for-profit	Private not-for-profit	Total CDs	Expected births	Rates
**National**						
2008	12,316	2864	1965	17,145	2,638,891	0.6%
2016	58,667	13,914	6335	78,916	2,928,303	2.7%
Tigray						
2008	1179	2	0	1181	160,929	0.7%
2016	5298	106	0	5404	163,802	3.3%
Afar						
2008	22	0	0	22	52,634	0.0%
2016	133	0	96	229	56,222	0.4%
Amhara						
2008	1503	28	0	1531	642,084	0.2%
2016	9993	533	437	10,963	660,518	1.7%
Oromia						
2008	2654	22	1000	3676	1,013,011	0.4%
2016	16,447	1042	1751	19,240	1,099,485	1.7%
Somali						
2008	104	8	0	112	165,580	0.1%
2016	488	39	0	527	178,048	0.3%
Benishangul-Gumuz						
2008	166	0	0	166	25,023	0.7%
2016	700	0	0	700	32,913	2.1%
SNNP						
2008	1822	100	536	2458	561,086	0.4%
2016	10,258	98	2507	12,863	595,296	2.2%
Gambella						
2008	76	0	0	76	11,448	0.7%
2016	135	0	0	135	13,420	1.0%
Harari						
2008	648	0	0	648	6545	9.9%
2016	915	381	0	1296	7568	17.1%
Addis Ababa						
2008	3918	2607	429	6954	97,755	7.1%
2016	13,243	11,351	1544	26,138	106,625	24.5%
Dire Dawa						
2008	224	97	0	321	12,239	2.6%
2016	1057	364	0	1421	14,405	9.9%

*SNNP* Southern Nations, Nationalities, Peoples’ region

At the national level, 72 and 74% of the cesareans were performed in public hospitals in 2008 and 2016, respectively. Half of the cesareans performed in Addis Ababa took place in the private for-profit and private not-for-profit hospitals. The CD rate in Addis Ababa was likely underestimated in 2008 due to the 12 private hospitals not eligible for the assessment.

The institutional CD rate is the percentage of institutional births delivered by cesarean and is influenced by patient mix and provider practice (Table 2). Patterns of institutional CD rates in 2008 and 2016 were similar: the private for-profit hospitals had the highest rates (46 and 54%, respectively) and the public hospitals had the lowest (15 and 20%, respectively), while the private not-for-profit hospital rates fell between the other two groups. The percentage increase among public hospitals was 29, 18% among private for-profit hospitals, and 7% among private not-for-profit hospitals.

**Table 2:** Absolute numbers of cesarean deliveries (CDs) and institutional deliveries and CD rates among hospitals that performed CDs in the last 3 months, by managing authority and year of assessment

Managing Authority	2008			2016		
	CDs	Institutional deliveries	Institutional CD rate	CDs	Institutional deliveries	Institutional CD rate
Public	12,316	80,135	15.4%	54,983	290,150	18.9%
Private for-profit	2864	6212	46.1%	13,810	25,384	54.4%
Private not-for-profit	1965	7342	26.8%	6335	22,523	28.1%
Total	17,145	93,689	18.3%	75,128	338,057	22.2%

### Hospital readiness to perform cesarean delivery

Approximately 80% of hospitals in both time periods provided CD services in the 3 months prior to the assessments (Table 3). Among hospitals that had performed CDs, the availability of surgeons, obstetricians and medical doctors declined between 2008 and 2016 when emergency surgical officers (ESOs) began to play a major role, especially in public hospitals. As their name suggests, ESOs are associate clinicians with a three-year master’s level of training to conduct common operations such as obstetric surgery, exploratory laparotomy and appendectomy. The cadre was designed to remedy the shortage of obstetrician/gynecologists and surgeons and it recruits from among BSc health officers and BSc nurses. The private for-profit hospitals relied almost entirely on obstetricians, while private not-for-profit hospitals increased their reliance on both obstetricians and ESOs. An increase from 95 to 100% among all hospitals performing CDs reported at least one professional on staff to administer anesthesia.

**Table 3:** Percent of hospitals that provided cesarean delivery in the last 3 months, and among those, characteristics of readiness, by managing authority and year of assessment

	Total number of hospitals		Managing authority					
			Public		Private for-profit		Not-for-profit	
	2008	2016	2008	2016	2008	2016	2008	2016
	*n* = 111	*n* = 316	*n* = 90	*n* = 236	*n* = 15	*n* = 61	*n* = 6	*n* = 19
Performed CD in last 3 months	78%	80%	73%	80%	100%	85%	100%	68%
	*n* = 87	*n* = 253	*n* = 66	*n* = 188	*n* = 15	*n* = 52	*n* = 6	*n* = 13
**Hospital has HR for surgery**^**a**^								
Ob/gyn	72%	50%	68%	36%	100%	96%	50%	77%
General surgeon	39%	9%	41%	9%	27%	4%	67%	46%
Medical doctor	29%	6%	35%	6%	7%	2%	17%	8%
ESOs	na	63%	na	80%	na	6%	na	39%
Health officer	9%	2%	11%	2%	0%	0%	17%	0%
Midwife	3%	0%	3%	1%	7%	0%	0%	0%
Nurse	2%	0%	3%	1%	0%	0%	0%	0%
At least one person can provide	98%	99%	97%	100%	100%	96%	100%	100%
**Hospital has HR for anesthesia**^**a**^								
Anesthesiologist/anesthetist	91%	na	91%	na	87%	na	100%	na
Anesthesiologist	na	13%	na	10%	na	25%	na	8%
Anesthetist	na	95%	na	94%	na	96%	na	100%
Other professional^b^	30%	3%	29%	3%	33%	2%	33%	8%
At least one person can provide	95%	100%	95%	100%	93%	100%	100%	100%
**Hospital has anesthesia and equipment**								
Anesthetic vaporizer	79%	88%	79%	85%	87%	96%	67%	100%
Halothane	84%	87%	83%	86%	87%	89%	83%	85%
Ketamine	94%	85%	94%	84%	93%	90%	100%	85%
Lignocaine/lidocaine 4%^c^	95%	19%	97%	18%	87%	15%	100%	46%
Bupivacaine	na	76%	na	73%	na	83%	na	85%
Oxygen cylinder, manometer, flowmeter, tubes, connectors	94%	98%	94%	98%	100%	96%	83%	100%
Operating table	97%	100%	96%	100%	100%	100%	100%	100%
Adjustable shadowless overhead light	90%	94%	86%	92%	100%	100%	100%	100%
**Met readiness requirements**^**d**^	72%	86%	70%	85%	87%	97%	67%	68%
**OT with electricity at visit**^**e**^	99%	92%	100%	94%	100%	94%	83%	100%
**OT with water at visit**	99%	89%	99%	86%	100%	94%	100%	100%

*na* not applicable/not included in 2008, *CD* cesarean delivery, *ob/gyn* obstetrician/gynecologist, *HR* human resources, *OT* operating theater, *ESOs* emergency surgical officers

^a^ The 2016 human resource module was worded differently from 2008 and additional questions were used

^b^ Ob/gyns, pediatricians, medical doctors, general surgeons, ESOs, health officers and nurses

^c^ In 2008, the question asked about lidocaine 1 or 2%

^d^ Defined as [at least 1 professional to conduct operation + 1 for anesthesia] AND [EITHER an anesthesia machine + halothane or ketamine OR regional anesthesia (lignocaine/lidocaine 4% or bupivacaine)] AND [an oxygen cylinder with manometer, flowmeter, tubes and connectors + an operating table + overhead light]

^e^ In 2008, the question referred to functioning power in the facility on day of interview, not the OT

The availability of drugs and equipment did not change dramatically between the two assessments. In 2016, public hospitals were at some disadvantage compared to private for-profit and private not-for-profit hospitals. For example, in 2016, 85% of public hospitals had vaporizers compared to 96 to 100% among the two groups of private hospitals.

Based on the readiness requirements, hospital readiness increased in public hospitals from 70 to 85% and among private for-profit hospitals from 87 to 97%, but only 67–68% of private not-for-profit hospitals were staffed and equipped to provide CDs.

We also examined whether hospitals experienced interruptions in electricity and running water. In 2008 virtually all hospitals that regularly performed cesareans had functioning water and electricity in the operation theater or in the hospital itself on the day of the assessment. In 2016 the percentage dropped to closer to 90% for both electricity and water.

### Chart reviews: quality of clinical care and record-keeping

In 2008, 95 hospitals provided a total of 275 chart reviews while in 2016, 288 hospitals provided 568 cases. The average age of the women was 26 years in both assessments. However, women whose cesareans were performed in the private for-profit hospitals were on average 28 years of age. In 2016 nearly half (46%) of the women attending public hospitals were nulliparous, while only 25–28% of women attending private hospitals were nulliparous.

In both EmONC assessments, more than 75% of women whose CD was reviewed had an emergency cesarean (Table 4). As a proportion of reviewed CDs, emergency cesareans were least frequent in the private for-profit settings (47% in 2008 and 53% in 2016); emergency cesareans in public and private not-for-profit hospitals ranged from 83 to 85% in 2008 and to 90–91% in 2016. Proportionally, about three to four times as many women with a previous cesarean or uterine scar were seen in the two groups of private hospitals compared to public hospitals. This pattern repeated itself in 2016. Indications for CD did not change dramatically although the proportion of CPD/prolonged labors as an indication increased from 32 to 45% and breech presentation as an indication decreased from 13 to 3%.

**Table 4:** Percent distribution of cesarean deliveries reviewed according to indication, by managing authority and year of assessment

Type and indication for cesareans	All cesareans reviewed		Managing authority					
			Public		Private for-profit		Not-for-profit	
	2008	2016	2008	2016	2008	2016	2008	2016
	*n* = 275	*n* = 566	*n* = 209	*n* = 409	*n* = 45	*n* = 125	*n* = 21	*n* = 32
**Type of cesarean**								
Emergency	77%	82%	83%	90%	47%	53%	85%	91%
Elective	21%	12%	15%	6%	51%	32%	15%	6%
No information	2%	6%	2%	4%	2%	15%	0%	3%
**Maternal indications**								
CPD/prolonged labor^a^	34%	45%	36%	50%	27%	28%	26%	40%
Previous cesarean	11%	13%	7%	8%	27%	26%	21%	22%
Placenta praevia/abruption	7%	6%	7%	7%	2%	4%	16%	3%
Severe PE/E	6%	4%	7%	4%	4%	2%	0%	6%
Other maternal indications^b^	9%	5%	10%	3%	8%	11%	0%	5%
**Fetal indications**								
Fetal distress^c^	15%	13%	13%	14%	20%	11%	21%	12%
Breech	14%	3%	17%	4%	2%	2%	11%	3%
Cord prolapse	2%	2%	3%	2%	0%	2%	0%	3%
Multiple gestation	0%	3%	0%	3%	0%	2%	0%	0%
Other fetal indications^d^	2%	3%	1%	2%	8%	4%	5%	6%
No information	0%	4%	0%	3%	2%	7%	0%	0%

^a^ Cephalo-pelvic disproportion, malpresentation, prolonged 1st and 2nd stages of labor, arrest disorders, failure to progress, failed assisted vaginal delivery, failed induction, and uterine rupture

^b^ Failed induction and assisted vaginal delivery, fistula, medical disease, maternal request and trauma

^c^ Fetal distress, severe intrauterine growth restriction and non-reassuring biophysical state

^d^ Meconium stained amniotic fluid, post-term, intrauterine fetal death, HIV+ mother and premature rupture of membranes

Information related to the quality of the clinical management of CDs is found in Table 5. In 2016, 50% of the women in the public hospitals whose cases were reviewed received prophylactic uterotonics after the baby was delivered. The percentage was 46% among women in private for-profit hospitals and 41% in the private not-for-profit sector. The use of prophylactic antibiotics was higher, with an overall increase from 87% in 2008 to 94% in 2016.

**Table 5:** Percent or percent distribution of cesarean deliveries reviewed according to administration of prophylactic uterotonics and antibiotics, use of partograph, time interval between decision and operation, type of anesthesia and practitioner and fetal outcomes, by managing authority and year of assessment

	All cesareans reviewed		Managing authority					
			Public		Private-for-profit		Not-for-profit	
	2008	2016	2008	2016	2008	2016	2008	2016
	*n* = 275	*n* = 568	*n* = 209	*n* = 409	*n* = 45	*n* = 127	*n* = 21	*n* = 32
**Prophylactic uterotonics administered after baby delivered**	na	50%	na	52%	na	46%	na	41%
**Prophylactic antibiotics administered**^**a**^	87%	94%	86%	94%	93%	91%	85%	94%
**Partograph use among women with non-elective cesareans**	*n* = 211	*n* = 499	*n* = 172	*n* = 383	*n* = 22	*n* = 86	*n* = 17	*n* = 30
Partograph used	11%	35%	9%	42%	0%	6%	47%	27%
Partograph not used	88%	55%	90%	49%	100%	78%	53%	60%
Planned elective went into labor	na	6%	na	5%	na	9%	na	7%
No information	1%	4%	1%	4%	0%	7%	0%	6%
**Time interval between decision and operation**^**b**^	*n* = 82	*n* = 148	*n* = 64	*n* = 130	*n* = 11	*n* = 9	*n* = 7	*n* = 9
Mean (minutes)	310	210	277	202	441	308	289	232
Median (minutes)	56	76	60	80	45	60	30	45
**Type of anesthesia used**								
Spinal/epidural (1 case)	na	59%	na	60%	na	49%	na	81%
General	na	28%	na	24%	na	36%	na	9%
Ketamine	na	4%	na	5%	na	2%	na	6%
No information	na	9%	na	8%	na	13%	na	3%
**Type of surgeon**								
Ob/gyn	na	41%	na	25%	na	88%	na	69%
General surgeon	na	31%	na	38%	na	7%	na	25%
ESO	na	21%	na	29%	na	0%	na	3%
Health officer	na	3%	na	4%	na	0%	na	0%
General practitioner	na	1%	na	2%	na	0%	na	3%
MSC midwife	na	1%	na	1%	na	0%	na	0%
No information	na	2%	na	1%	na	5%	na	0%
**Fetal Outcomes**	*n* = 275	*n* = 566	*n* = 209	*n* = 409	*n* = 45	*n* = 125	*n* = 21	*n* = 32
Live birth	79%	95%	78%	94%	93%	93%	71%	91%
Stillbirth	9%	2%	11%	3%	2%	2%	5%	3%
Neonatal death	5%	< 1%	5%	0%	4%	0%	5%	0%
Live birth + death	2%	< 1%	2%	0%	0%	0%	0%	0%
No information	5%	3%	5%	2%	0%	6%	19%	6%

*na* not applicable/not included in 2008, *ESO* emergency surgical officer, *MSC* BSc midwife with surgical training

^a^ In 2008, the timing of antibiotics was not specified. In 2016, timing reflects antibiotics given either pre- or post-operatively. If no documentation of antibiotics was recorded, we assumed the woman did not receive them

^b^ Asked of emergency cesarean deliveries only

Among women whose cesareans were an emergency, defined by having gone into labor, partograph use increased overall from 11 to 35%, occurring almost exclusively in public hospitals (9 to 42%).

We analyzed the time from decision to surgery for women with emergency cesareans, despite a high rate of missing information (58% in 2008 and 66% in 2016). The median interval in 2008 was 56 min; it increased to 75 min in 2016. Increases took place across all sectors.

Questions regarding the type of anesthesia administered and the cadre of the surgeon were asked only in 2016. Type of anesthesia varied widely across hospital groups with 81% of reviewed cases in private not-for-profit hospitals using spinal anesthesia, compared to 49% in private for-profit hospitals and 60% among cases in public hospitals. General anesthesia was the second most reported type of anesthesia across all hospitals. In public hospitals, 25% of women had an obstetrician in attendance, 39% had a general surgeon and 29% an emergency surgical officer. In the private for-profit hospitals, 88% of women were attended by an obstetrician, an additional 7% had a general surgeon, and no cases were attended by an emergency surgical officer. In the not-for-profit hospitals the percentages were 69% (obstetrician), 25% (general surgeon) and 3% (emergency surgical officer).

Fetal outcomes improved over time with live births increasing from 80 to 94%. The improved fetal outcomes were most evident in private not-for-profit and public hospitals. Maternal deaths among the reviewed cases declined from two in 2008 to one in 2016.

### Robson classification

Figure 1 shows the relative contribution of each Robson group to all CDs reviewed by managing authority. In public hospitals, Robson group 1 accounted for 33% of the cesareans reviewed, followed by group 8 (12%). In the private for-profit hospitals, group 5 dominated (28%), followed by group 2 (17%). This aligns with the findings on indications (Table 4), in which “previous cesarean” was the second most frequent indication in private for-profit hospitals. Forty-two percent of the reviewed cesareans from the private not-for-profit hospitals were women belonging equally to groups 1 and 5. As stated earlier, about a third of the cases suffered missing information and could not be grouped.

## Discussion

Nationally, Ethiopia exhibits a large unmet need for cesarean delivery. When disaggregated by region, CD rates ranged from 24% in Addis Ababa to under 1% in pastoralist regions. Rates were highest in highly urbanized regions where private for-profit hospitals contributed between 25 and 50% of all cesareans. Although public hospitals provided the bulk of obstetric services, by 2016 more than half (54%) of all deliveries in private for-profit hospitals were cesareans. Private for-profit hospitals exhibited the highest elective CD rate. Hospital readiness to perform a cesarean showed signs of improvement, and a larger proportion of hospitals overall met minimum staffing and equipment requirements in 2016 than in 2008. Private for-profit hospitals demonstrated the lowest partograph use (6%) while partograph use increased from 9 to 42% in public hospitals and declined among private not-for-profit hospitals from 47 to 27%. In 2016, 40% of CD reviews from public hospitals were among low-risk nulliparous women (Robson groups 1&2) and 45% of chart reviews at private for-profit hospitals consisted of Robson groups 2 and 5.

Despite the tripling of the overall number of hospitals between 2008 and 2016, both assessments indicated that about four out of five hospitals provided CD services, perhaps indicating an opportunity to increase access with minimal investment. The lack of universal water and electricity in hospitals in 2016 might have been a result of the rapid expansion in infrastructure. One enabling factor for improvements in hospital readiness was likely the establishment in 2009 of the emergency surgical officer [21]. Four out of five public hospitals with CD services employed ESOs. A large push to prepare anesthetists also took place during this time period [21].

Chart reviews revealed how patient profiles and provider practices varied by managing authority. The private for-profit and private not-for-profit hospitals disproportionately attracted women with a previous CD and non-emergency cases. The chart reviews also pointed to mixed results regarding the practice of evidence-based interventions. The use of partographs to monitor labor more than tripled in public hospitals while the partograph was hardly used or declined elsewhere. Uterotonics administered after the baby was delivered and prophylactic antibiotic use were also more evident in public hospitals than elsewhere. These three interventions can help prevent serious complications and all women who undergo a cesarean delivery should receive them. Thus, it is encouraging that contrary to other experiences [25], public hospitals performed as well if not better than private hospitals.

The inclusion of the Robson classification parameters in 2016 enabled us to see clearly the perpetuation of “once a cesarean, always a cesarean,” especially among the women who delivered in private for-profit and private not-for-profit hospitals given the contribution of group 5 to these two groups of hospitals.

Typically, groups 1, 2, 3, 4 and 5 contribute heavily to the overall CD rate, while groups 6–10 account for a smaller proportion of all cesareans. According to a WHO multi-country study from 21 countries and 287 hospitals, groups 6–10 accounted for only 20% of the cesareans [26]. In our case, groups 6–10 accounted for 38% of the cases. This might reflect Ethiopia’s overall low cesarean rate and that higher risk women contributed more to the pool of cesareans than in the WHO multicenter study. As the overall cesarean rate in Ethiopia increases, the contribution of groups 6–10 relative to other groups may decline. Nevertheless, it is worrisome that 40% of the chart reviews at public hospitals were among low risk nulliparous women. One of the goals of this classification system is to reduce cesareans among groups 1 and 2, who are known to be vulnerable to unnecessary CDs, and who are often the biggest group as we observed in this study [26]. Overuse of CD among these groups sets up a domino effect that contributes to repeated cesareans.

The two extremes – widespread underutilization of life-saving surgical care and the overutilization of a costly procedure not without risk – is an opportunity for Ethiopia, and other low and middle income settings faced with similar dual challenges, to improve availability and access to quality CD care and general coverage by elevating rates where needed, while learning from high income regions’ mistakes of overusing cesarean delivery.

### Strengths and limitations

A strength of this paper is the richness of its data sources: two national health facility censuses that enabled comparisons between public and private managing authorities. Analyses drew on data from interviews with health workers, observation of infrastructure, 12 months of service statistics, and individual level data from women who had undergone a cesarean, resulting in a multifaceted overview of how rates, hospital readiness and quality of clinical management of cesarean delivery changed as well as suggesting some steps going forward. Results regarding the specifics of hospital readiness are highly generalizable since all or nearly all hospitals were included in the assessments.

We recognize that observation generally produces more accurate results than reporting, especially if recall is required. Hospital readiness depended heavily on responses from staff rather than observation, for example, whether an oxygen cylinder was both available and functional. Furthermore, readiness results will change as do the requirements, and we encourage strengthening the definition of readiness.

Data quality of the primary sources – admission, operating theater and discharge logbooks – is a well-recognized limitation when working with health facility data. We are not clear why an increase in missing information occurred between the two assessments, especially among the case reviews. The inability to classify a third of the cesareans reviews into one of the 10 Robson groups points to omissions in record-keeping, even though the six parameters used to group women are considered standard data items. The group that suffered the least amount of missing information (20 cases) was group 8, defined only by singleton or multiple gestation. Group 5 suffered the most from missing information: 132 of 568 cases lacked at least one of the five variables that defined group 5, with “previous cesarean” the most frequently missing.

The chart reviews were systematically selected but were technically a convenience sample, and do not represent all cesareans, especially those conducted in the private for-profit hospitals where missing data tended to be the highest. Similar studies have struggled with the use of non-randomly selected chart reviews but also recognized their value when presenting an overview of a single service delivery intervention [27].

Some variables were only available in 2016 and the rewording of some questions affected our ability to make precise comparisons for other variables, but periodic surveys often undergo modifications in their data collection instruments, which are intended to enhance the data quality over time.

## Conclusions

During the 8-year interval between assessments, the government tripled the number of hospitals, raised the CD delivery rate in underserved regions, and improved both the readiness to perform obstetric operations and the quality of clinical care. The data also point to dangerously low CD rates in underserved rural areas. More equitable access to cesarean services can be achieved through strengthened referral systems, hospital maternity waiting homes and the continued expansion of facilities that fill geospatial gaps [28, 29]. Meanwhile, high institutional CD rates in the private sector, especially private for-profit hospitals, suggest that not all cesareans may be medically indicated. Federal normative bodies as well as professional societies should investigate local conditions to identify what is driving the demand: financial incentives on the supply side, professional inexperience with non-routine vaginal births, or other forces. The use of the Robson classification would be a useful tool for future quality improvement efforts – to ensure that the right women are receiving this procedure while others are protected from unnecessary procedures.

## Supplementary Information

**Additional file 1: Module 1.** Identification of facility and infrastructure.

**Additional file 2: Module 2.** Human resources.

**Additional file 3: Module 3.** Essential Drugs, Equipment, and Supplies.

**Additional file 4: Module 4.** Facility Case Summary.

**Additional file 5: Module 5.** EmOC and EmNeC Signal Functions and Other Essential Services.

**Additional file 6: Module 8.** Cesarean Delivery Review.

## Data Availability

The datasets generated and analyzed for this study are available from the Ethiopian Public Health Institute Director on reasonable request.
