# Supplementary material for: Cesarean delivery rates, hospital readiness and quality of clinical management in Ethiopia: national results from two cross-sectional emergency obstetric and newborn care assessments
Source: BMC Pregnancy Childbirth. 2021 Aug 19;21:571. doi: 10.1186/s12884-021-04008-9 (PMC8377989; doi:10.1186/s12884-021-04008-9)
Supplement: Supplementary file 1 — Additional file 1: Module 1. Identification of facility and infrastructure. [file 12884_2021_4008_MOESM1_ESM.doc]

EmONC Assessment

MODULE 1: Identification of Facility and Infrastructure

**Interviewer Name:**

***INSTRUCTIONS****:* *The data collection team leader should complete this section as soon as the team arrives at the facility and before interviewing the facility officer in charge. Copy the Facility ID (FID) onto each page of each Module before the team begins to collect data.*

| Number | Question | | | Result | | | |
| --- | --- | --- | --- | --- | --- | --- | --- |
| SECTION 1: COVER PAGE | | | | | | | |
| Date | QDAY  _______________  Day | | QMONTH  _______________  Month | | QYEAR  _____________  Year | |  |
| FACILITY IDENTIFICATION | | | | | | | |
| 005 | Region/City administration code | |  | | | | |
| 006 | Zone/sub-city code | |  | | | | |
| 001 | Facility ID | |  | | | | |
| 003 | Official name of facility | |  | | | | |
| ETH_01 | Woreda Name | |  | | | | |
| ETH_02 | Town Name | |  | | | | |
| ETH_03 | Kebele Name | |  | | | | |
| QINTERVIEWER | Interviewer Code | |  | | | | |
| 007 | Type of facility | | REFERRAL/SPECIALIZED HOSPITAL  GENERAL HOSPITAL  PRIMARY HOSPITAL  HEALTH CENTRE  MCH SPECIALIZED CENTER  MCH SPECIALIZED CLINIC  HIGHER CLINIC  OTHER (*SPECIFY*) _________________ | | | 1  2  3  4  5  6  7  96 | |
| 008 | Managing Authority | | GOVERNMENT/PUBLIC  NGO/NOT-FOR-PROFIT  PRIVATE-FOR-PROFIT  MISSION/FAITH-BASED  OTHER (*SPECIFY*) | | | 1  2  3  4  96 | |
| 009 | Urban/Rural | | URBAN  RURAL | | | 1  2 | |
| 010 | Facility Operational status | | FULLY FUNCTIONAL: NO CONTRUCTION OR EXPANSION  FULLY FUNCTIONAL: CURRENTLY UNDER EXPANSION  PARTIALLY FUNCTIONAL: CURRENTLY UNDER EXPANSION  NOT FUNCTIONAL: CURRENTLY UNDER CONSTRUCTION  NOT FUNCTIONAL: REASON UNKNOWN | | | 1  2  3  4  5 | |
| GEOGRAPHIC COORDINATES | | | | | | | |
| SET DEFAULT SETTINGS FOR GPS.  STAND IN A LOCATION AT THE ENTRANCE OF THE FACILITY WITH PLAIN VIEW OF THE SKY.  INSERT GPS DONGLE IN TABLET USB PORT AND …. | | | | | | | |
| 011 | Waypoint name  (Facility ID) |  | | | | | |
| 012 | Altitude | Meters | | | | | |
| 013 | Latitude | 1. N/S 2. DEG/MIN/SEC | | | | | |
| 014 | Longitude | 1. N/S 2. DEG/MIN/SEC | | | | | |
| FACILITY VISUAL IDENTIFICATION | | | | | | | |
| TAKE 2 PHOTOS OF THE FACILITY AS PER THE INSTRUCTION BELOW:  PHOTO 1: PHOTO OF THE NAME OF THE HEALTH FACILITY, EITHER THROUGH CLOSE UP OF SIGN OR HANDWRITTEN ON PAPER  PHOTO 2: STAND 20 TO 30 METERS BACK FROM CENTER OF ENTRANCE GATE OUTSIDE THE COMPOUND BUT FACING THE COMPOUND  IN CASE PHOTOS CANNOT BE TAKEN OUTSIDE THE COMPOUND, THEN STAND AS CLOSE AS POSSIBLE TO THE FENCE IN THE SAME POSITION AS DESCRIBED ABOVE. | | | | | | | |

Q15 May I proceed with the interview? 1. Yes 0. No

Q16A Hour interview began ___ ___

Q16B Minutes interview began ___ ___

SECTION 1. Facility Identification

**INSTRUCTIONS**: *Direct these questions to the officer in charge.*

| **No.** | **Item** | **Response** |
| --- | --- | --- |
| Q101_1 | Since the major topic of interest of this survey is obstetric and newborn care, could you tell us if any deliveries have been attended in this facility in the last 12 months? | Yes. 1  No 0  *(If “No,” please immediately inform your team members that there have been no deliveries in the last 12 months and terminate the interview and the visit.)* |
| Q102A_1 | Which month and year was the last delivery? | Month |___|___| Year |___|___|___|___| |
| Q102B_1 | Which month and year was the facility started providing delivery services?  *(88 8888=Don’t know)* | Month |___|___| Year |___|___|___|___| |
| Q106_1 | Does this facility have a specified catchment area—that is, a defined geographic or administrative area for which the facility has direct responsibility for serving? | Yes. 1  No 0  *(If “No,” skip to section 2)* |
| Q107_1 | How many people are supposed to be in the catchment area for this facility?  *(88 888 888 = don’t know)* | |___|___|___|___|___|___|___|___| |

SECTION 2. Facility Infrastructure

I’d like to ask you a few questions about the facility’s overall capacity and infrastructure.

| **No.** | **Item** | **Response** | | | **Skip to** |
| --- | --- | --- | --- | --- | --- |
| Q201_1 | How many beds are available for patients in this facility (total in all departments)?  *(write number)* | |___|___|___| | | |  |
| Q202_1 | How many beds are dedicated exclusively for:   1. obstetric and gynecological patients (antenatal, postpartum, post-op, post abortion, etc.) 2. patients in the 1^st^ stage (labor beds) 3. patients in the 2^nd^ stage (delivery couches) | 1. |___|___|___| 2. |___|___|___| 3. |___|___|___| | | |  |
| Q203_1 | Does this facility get electricity from the electricity grid (central supply)?  *(even if irregular,  circle 1 for “Yes”)* | Yes 1  No 0 | | |  |
| Q205_1 | Does this facility have other sources of electricity? | Yes 1  No 0 | | | If “No,” skip to Q204_1 |
| Q206_1 | What other sources of electricity does the facility have?  *(read items)*  a. Generator (fuel operated)  b. Generator (battery operated)  c. Solar  d. Other *(specify)*  __________________________ | Yes  1  1  1  1 | | No  0  0  0  0 | If no type of generator indicated, skip to Q204_1 |
| Q207_1 | Is the generator functional, in other words, if you needed it today, could you use it? | Yes 1  No 0  Don’t know 8 | | |  |
| Q208_1 | Does the generator come on automatically if there is a loss in power? | Yes 1  No 0  Don’t know 8 | | |  |
| Q204_1 | Thinking back over the last 7 days, has the power from the grid or any other source been interrupted for more than 2 hours at a time? | Interruption >2 hr 1  Interruption <2 hr 2  No interruption 0  No source of electricity 9 | | |  |
| Q209_1 | Does this facility have water for its basic functions? | Yes 1  No 0 | | | If “No,” skip to Q217_1 |
| Q210_1 | What is the *most commonly used* source of water?  *(circle one)* | Piped water 1  Hand pump 2  Well 3  River 4  Other (*specify*) 96  ________________________ | | |  |
| Q211_1 | Is the water from this source onsite, within 500 meters of the facility, or beyond 500 meters of the facility? | Onsite/within the compound 1  Within 500 m of facility 2  Beyond 500 m of facility 3 | | |  |
| Q212_1 | In the past 3 months, has this facility ever been without water for a full 24 hours? | Yes 1  No 0 | | |  |
| Q213_1 | Does the facility have:  (*read options)* | Yes | No | | If “Yes” water tank, continue to Q214_1. All other answers skip to Q217_1 |
|  | 1. A dug well? | 1 | 0 | |  |
|  | 1. A water tank? | 1 | 0 | |  |
| Q214_1 | What is the maximum volume of the water tank?  88888 = don’t know | |___|___|___|___|___| liters | | |  |
| Q215_1 | What is the primary source of water for filling the tank? | Harvested rain 1  Truck/vessel/”Botae” 2  Piped water 3  Other *(specify)* 96  ________________________ | | |  |
| Q216_1 | With what frequency is the tank filled? | Weekly 1  Every 2 weeks 2  Monthly 3  Other (*specify*) 96  ________________________  Don’t know 8 | | |  |
| Q217_1 | Is there a toilet (latrine) in functioning condition for general **staff use**? | Yes 1  No 0 | | | If “No,” skip to  Q219_1 |
| Q218_1 | What type of toilet or latrine is it? | Flush or pour flush toilet 1 | | |  |
|  |  | Ventilated improved pit latrine 2 | | |  |
|  |  | Pit latrine with slab 3 | | |  |
|  |  | Pit latrine without slab/open pit 4 | | |  |
|  |  | Composting toilet 5 | | |  |
|  |  | Other *(specify)* 96 | | |  |
|  |  | Don’t know 8 | | |  |
| Q219_1 | Is there a toilet (latrine) in functioning condition for **patient use**? | Yes 1  No 0 | | | If “No,” skip to  Q221_1 |
| Q220_1 | What type of toilet or latrine is it? | Flush or pour flush toilet 1 | | |  |
|  |  | Ventilated improved pit latrine 2 | | |  |
|  |  | Pit latrine with slab 3 | | |  |
|  |  | Pit latrine without slab/open pit 4 | | |  |
|  |  | Composting toilet 5 | | |  |
|  |  | Other (*specify)* 96 | | |  |
|  |  | Don’t know 8 | | |  |

*INSTRUCTIONS: For each physical area below ask if the facility has a separate room, for example, for ANC. If the answer is “yes,” ask if electricity and water are functioning in the room at the time of the visit. If there is no separate room, for example, antenatal care, circle “0” in column “a,” skip columns “b” and “c,” and continue with the next row.*

| **No.** | **Physical areas** | **a. Does this facility have a (separate) room/space for…?** | | **b. Is electricity functioning at the time of this visit in ….?** | | **c. Is there a source of water at the time of this visit in ….?** | |
| --- | --- | --- | --- | --- | --- | --- | --- |
|  | *Read each item:* | Yes | No | Yes | No | Yes | No |
| Q221_1 | Antenatal care | 1 | 0 | 1 | 0 | 1 | 0 |
| Q222_1 | Labor and delivery together | 1 | 0 | 1 | 0 | 1 | 0 |
| Q223_1 | Labor (first stage) | 1 | 0 | 1 | 0 | 1 | 0 |
| Q224_1 | Delivery | 1 | 0 | 1 | 0 | 1 | 0 |
| Q225_1 | Maternity room for pregnancy complications, e.g. pre-eclampsia/ eclampsia | 1 | 0 | 1 | 0 | 1 | 0 |
| Q226_1 | Postnatal room | 1 | 0 | 1 | 0 | 1 | 0 |
| Q227_1 | Post C/S ward | 1 | 0 | 1 | 0 | 1 | 0 |
| Q228_1 | General operating theatre | 1 | 0 | 1 | 0 | 1 | 0 |
| Q229_1 | Ob/gyn operating theatre | 1 | 0 | 1 | 0 | 1 | 0 |
| Q230_1 | Intensive Care Unit | 1 | 0 | 1 | 0 | 1 | 0 |
| Q231_1 | Newborn corner | 1 | 0 | 1 | 0 | 1 | 0 |
| Q232_1 | Neonatal intensive care unit (NICU) | 1 | 0 | 1 | 0 | 1 | 0 |
| Q233a_1 | Pediatric ward | 1 | 0 | 1 | 0 | 1 | 0 |
| Q233b_1 | IMNCI clinic | 1 | 0 | 1 | 0 | 1 | 0 |
| Q234_1 | Laboratory and mini blood bank together | 1 | 0 | 1 | 0 | 1 | 0 |
| Q235_1 | Laboratory | 1 | 0 | 1 | 0 | 1 | 0 |
| Q236_1 | Separate mini blood bank | 1 | 0 | 1 | 0 | 1 | 0 |

SECTION 3. Facility Service Delivery

| **No.** | **Item** | **Response** | |
| --- | --- | --- | --- |
| Q301_1 | We’d like to know about some of the basic services provided at this facility. Does the facility provide:  *(read each item)* | **Yes** | **No/NA** |
|  | 1. Focused antenatal care | 1 | 0 |
|  | 1. Postnatal care | 1 | 0 |
|  | 1. Cervical screening (pap smear or VIA single visit approach) | 1 | 0 |
|  | 1. Family planning services | 1 | 0 |
|  | 1. Diagnosis and treatment for sexually transmitted infections (STIs) | 1 | 0 |
|  | 1. Adolescent/youth friendly sexual and reproductive health services | 1 | 0 |
|  | 1. PMTCT of HIV package | 1 | 0 |
|  | 1. Anesthesia for major surgery | 1 | 0 |
|  | 1. Blood typing services | 1 | 0 |
|  | 1. Normal delivery | 1 | 0 |
|  | 1. Assisted vaginal delivery | 1 | 0 |
|  | 1. C/S delivery | 1 | 0 |
|  | 1. Blood transfusion | 1 | 0 |
|  | 1. Post-abortal care | 1 | 0 |
|  | 1. Safe abortion | 1 | 0 |

SECTION 4. Payment for Services

The next series of questions have to do with payment for services in general and for obstetric/gynecological emergencies.

| **No.** | **Item** | **Response** | | | **Skip to** |
| --- | --- | --- | --- | --- | --- |
| Q401_1 | Is there a formal payment required before receiving services / treatment? | Yes 1  No 0 | | |  |
| Q402_1 | Is a woman expected to pay a fee for the delivery of services? | Yes 1  No 0 | | |  |
| Q403_1 | Is a woman expected to pay for/buy supplies and medicines for delivery? | Yes 1  No 0 | | |  |
| Q404_1 | In an obstetric/gynecological emergency, is payment required before a woman can receive treatment? | Yes 1  No 0 | | |  |
| Q405_1 | In an obstetric/gynecological emergency, is the woman or her family asked to buy medicine or supplies prior to treatment? | Yes 1  No 0 | | |  |
| Q406_1 | Is there a fee schedule for services posted in a visible and public place?  *(by observation only)* | Yes 1  No 0 | | |  |
| Q407_1 | What is the approximate current cost to an obstetric patient in this facility (in birr) for:  (*read each item, and enter 0000.00 if there is no cost to the patient; 9999.99 if service or item is never available; 8888.88 if the respondent does not know)*  (NB: inj = injection) |  | | |  |
|  | 1. Card fee | 1. \|___\|___\|___\|___\| .\|__\|__\| | | |  |
|  | 1. Normal labor/delivery | 1. \|___\|___\|___\|___\| .\|__\|__\| | | |  |
|  | 1. Cesarean delivery | 1. \|___\|___\|___\|___\| .\|__\|__\| | | |  |
|  | 1. Assisted vaginal delivery (vacuum extraction or forceps) | 1. \|___\|___\|___\|___\| .\|__\|__\| | | |  |
|  | 1. Neonatal special care unit (per day) | 1. \|___\|___\|___\|___\| .\|__\|__\| | | |  |
|  | 1. Gloves | 1. \|___\|___\|___\|___\| .\|__\|__\| | | |  |
|  | 1. IV fluids | 1. \|___\|___\|___\|___\| .\|__\|__\| | | |  |
|  | 1. Prescription of oxytocin (inj) | 1. \|___\|___\|___\|___\| .\|__\|__\| | | |  |
|  | 1. Prescription of antibiotic | 1. \|___\|___\|___\|___\| .\|__\|__\| | | |  |
|  | 1. Prescription of magnesium sulfate (inj) | 1. \|___\|___\|___\|___\| .\|__\|__\| | | |  |
|  | 1. Safe abortion | 1. \|___\|___\|___\|___\| .\|__\|__\| | | |  |
|  | 1. Post abortion care | 1. \|___\|___\|___\|___\| .\|__\|__\| | | |  |
|  | 1. Laparotomy | 1. \|___\|___\|___\|___\| .\|__\|__\| | | |  |
| Q408_1 | Are women charged separately for the following things:  *(read each item)* | Yes | No | Not available |  |
|  | a. Bed | 1 | 0 | 9 |  |
|  | b. Food for mother | 1 | 0 | 9 |  |
|  | c. Blood transfusion | 1 | 0 | 9 |  |
|  | d. Formula milk | 1 | 0 | 9 |  |
| Q409_1 | Is there a **formal** system in place to have fees for maternity services waived for poor women? | Yes 1  No 0 | | | If “Yes,” skip to Section 5. |
| Q410_1 | Is there an **informal** system in place to have fees for maternity services waived for poor women? | Yes 1  No 0 | | |  |

SECTION 5. Policy Environment of Health Facility

| **No.** | **Item** | **Response** | | **Skip to** |
| --- | --- | --- | --- | --- |
| Q501_1 | How many hours do women generally stay at the facility following a normal delivery?  *(Convert into hours if answer is day(s))* | |___|___| hrs | |  |
| Q502_1 | How many hours on average do women stay at the facility following C/S delivery?  *(Convert into hours if answer is days)*  *999 = facility does not provide C/S* | |___|___|___| hrs | |  |
| Q503_1 | Does the facility carry out audits or case reviews of maternal deaths on a routine basis?  *(Routine basis: after every maternal death, or on a systematic, regular basis; e.g., every month or every 6 months.)* | Yes 1  No 0  Never had a death 9 | |  |
| Q504_1 | Has this facility implemented the Maternal Death Surveillance and Response (MDSR) initiative? | Yes 1  No 0 | | If “No,” skip to Q506_1 |
| Q505_1 | Has a Maternal Death Surveillance and Response (MDSR) committee been established in this facility? | Yes 1  No 0  Don’t know 8 | |  |
| Q506_1 | Does this facility register maternal deaths by cause?  *(check the register if unsure)* | Yes 1  No 0  Never had a death 9 | |  |
| Q507_1 | Does the facility carry out audits or case reviews for newborn deaths and/or stillbirths on a routine basis?  *(Routine basis: after every stillbirth and newborn death, or on a systematic, regular basis; e.g., every month or every 6 months.)* | No 0  Yes for both 1  Yes, but only newborn deaths 2  Never had a death 9 | |  |
| Q508_1 | Does the facility carry out near miss reviews on a routine basis?  *(Routine basis: after every near miss, or on a systematic, regular basis; e.g., every month or every 6 months.)* | Yes 1  No 0  Never had a near miss 9 | |  |
| Q509_1 | Does this facility have a policy that promotes frequent staff rotation (more than one rotation a year) to different areas of the facility? For example, for staff who provide:  *(read each item)* | Yes | No |  |
|  | 1. Maternal care | 1 | 0 |  |
|  | 1. Newborn care | 1 | 0 |  |
| Q510_1 | Does this facility allow a woman to have a female companion of her choice with her during:  *(read each item)* | Yes | No |  |
|  | a. labor? | 1 | 0 |  |
|  | b. delivery? | 1 | 0 |  |
| Q511_1 | Does this facility allow a woman to walk around during labor? | Yes 1  No 0  Don’t know 8 | |  |
| Q512_1 | Does this facility allow a woman to choose her preferred position during labor /delivery? | Yes 1  No 0  Don’t know 8 | |  |
| Q513_1 | Has this facility ever been certified by any mother-baby friendly birthing-facility initiative? | Yes 1  No 0  Don’t know 8 | |  |
| Q514_1 | Can a family register the birth of their baby at this facility in the government Vital Statistics and Civil Registration System? | Yes 1  No 0  Don’t know 8 | |  |
| Q515_1 | Has the government designated this facility an EmONC facility? | Yes 1  No 0  Don’t know 8 | | If “No” or “Don’t know”, skip to Section 6. |
| Q516_1 | Is the EmONC designation a B-EmONC (basic EmONC) or C-EmONC (comprehensive EmONC)? | Basic EmONC 1  Comprehensive EmONC 2  Other (*specify*) 96  _______________________ | |  |

| SECTION 6. HMIS | | | | |
| --- | --- | --- | --- | --- |
|  |  | | | |
| **No.** | **Item** | **Response** | | Skip to |
| Q601_1 | Does this facility have a system in place to regularly collect MNH services data? | Yes 1  No 0 | | If “No”, skip to Q 606_1 |
| Q602_1 | Does this facility regularly compile any reports containing MNH services? | Yes 1  No 0 | |  |
| Q603_1 | How frequently are these reports compiled? | Monthly 1  Quarterly 2  Other (*specify*) 96  _______________________ | |  |
| Q604_1 | Does this facility routinely calculate the following indicators: | Yes | No/NA |  |
|  | 1. Institutional delivery | 1 | 0 |  |
|  | 1. Institutional cesarean rate | 1 | 0 |  |
|  | 1. Institutional stillbirth rate | 1 | 0 |  |
|  | 1. Institutional low birth weight rate | 1 | 0 |  |
| Q605_1 | Does this facility have a data manager or HMIS staff who is responsible for data including MNH services data? | Yes 1  No 0 | |  |
| Q606_1 | Does this facility have a computer? | Yes 1  No 0 | |  |
| Q607_1 | Does the facility have internet access? | Yes 1  No 0 | |  |

| **Comments** |
| --- |
|  |
