# Supplementary material for: Cesarean delivery rates, hospital readiness and quality of clinical management in Ethiopia: national results from two cross-sectional emergency obstetric and newborn care assessments
Source: BMC Pregnancy Childbirth. 2021 Aug 19;21:571. doi: 10.1186/s12884-021-04008-9 (PMC8377989; doi:10.1186/s12884-021-04008-9)
Supplement: Supplementary file 2 — Additional file 2: Module 2. Human resources. [file 12884_2021_4008_MOESM2_ESM.doc]

## EmONC Assessment

MODULE 2: Human Resources

**Interviewer ID |___|___|___|___|**

**Date (dd/mm/yyyy): ____ / ____ / ________**

***INSTRUCTIONS:***

- *Direct questions under:*
  - ***Overall staffing*** *to the facility officer in charge or the administrator who works with payroll. If s/he does not know, go to the person in charge of the maternity.*
  - ***Emergency Obstetric and Newborn Signal Functions and Other Essential Services*** *and* ***24 Hour Availability*** *to the person in charge of the maternity. If s/he does not know who provides services in the operating theater, ask the person in charge of the operating theater at the time of the visit. These questions refer to services provided in this facility.*
- You should write the number of the answer to the first question (*How many are currently employed by this facility?*) for the first column (Medical doctor) and work vertically through the first section with reference to Medical Doctors. After you answer Q104_2, if the answer to Q101_2 for Medical Doctors is 0, skip to Q101_2 for the next cadre. If the answer to Q101_2 for Medical Doctors is 1 or more, continue to ask Q105_2 through Q110_2 for Medical Doctors. Then move on to the next category of health worker (Obstetrician/Gynecologist), and so on.
- For Section 2 “EmONC Signal Functions,” ask only about those professional groupings who currently work at the facility. Like the first section, Section 2 should be read from top to bottom for each type of health worker grouping.
- Include visiting medical personnel who are accredited and professional staff. Do not include students of any cadre in responses.
- **Medical residents and interns should be considered as medical doctors (general practitioners**). Do not include students of any cadre when asking the following questions.

SECTION 1. Overall Staffing (*write the number in each cell*)

| **No.** | | **Item** | **a. Medical doctor (general practitioner)** | **b. Obstetrician/**  **Gynecologist** | **c. General surgeon** | **d. Pediatrician** | **e. Neonatologist** | **f. Emergency**  **Surgical officer** | **g. Midwife BSC** | **h. Midwife diploma** | **i. Nurse BSC** | **j. Nurse diploma** | **k. Health officer** | **l. Anesthesiologist (MD**) | **m. Anesthetist BSC** | **n. Anesthetist diploma** | **o. Laboratory technician** | **p. Pharmacist /**  **Druggist** | **q. Health information technologist** |
| --- | --- | --- | --- | --- | --- | --- | --- | --- | --- | --- | --- | --- | --- | --- | --- | --- | --- | --- | --- |
| Q101_2 | | How many are currently employed by this facility? |  |  |  |  |  |  |  |  |  |  |  |  |  |  |  |  |  |
| Q102_2 | | How many established positions does this facility have for this type of staff member? |  |  |  |  |  |  |  |  |  |  |  |  |  |  |  |  |  |
| Q103_2 | | How many staff members left this facility in the last 12 months? |  |  |  |  |  |  |  |  |  |  |  |  |  |  |  |  |  |
| Q104_2 | | How many were hired/seconded to this facility in the last 12 months? |  |  |  |  |  |  |  |  |  |  |  |  |  |  |  |  |  |
| *For those cadres with at least one staff member currently employed at this facility (see 101_2), answer the remaining questions in Section 1. If this facility does not currently employ this cadre, you should not try to answer the remaining questions in Section 1 (rows 105_2 - 110_2) for those cadres.* | | | | | | | | | | | | | | | | | | | |
| Q105_2 | How many are currently on extended leave (more than 1 month)? | |  |  |  |  |  |  |  |  |  |  |  |  |  |  |  |  |  |
| Q106_2 | How many actually provide obstetric and newborn care? | |  |  |  |  |  |  |  |  |  |  |  |  |  |  |  |  |  |
| Q107_2 | Of those providing maternity care, how many have had training in BEmONC? | |  |  |  |  |  |  |  |  |  |  |  |  |  |  |  |  |  |
| Q108_2 | Of those providing maternity care, how many have had CEmONC training? | |  |  |  |  |  |  |  |  |  |  |  |  |  |  |  |  |  |
| Q109_2 | Of those providing maternity care, how many have had NICU training? | |  |  |  |  |  |  |  |  |  |  |  |  |  |  |  |  |  |
| Q110_2 | Of those providing maternity care, how many have had training in Essential Newborn Care / Helping Babies Breathe? | |  |  |  |  |  |  |  |  |  |  |  |  |  |  |  |  |  |

Go to Section 2 and circle or mark each category that has at least one staff member currently working in this facility (101_2). This should help you remember **to only ask questions** about professionals who are currently working in this facility. Medical residents and interns should be considered as medical doctors (general practitioners). Do not include students of any cadre when asking the following questions.

SECTION 2. Emergency Obstetric and Newborn Signal Functions and Other Essential Services

**Do staff from these groups of health workers provide the following services?** *(Provision of services should reflect real circumstances and not whether the health worker is authorized or formally trained to provide the care. Exclude health workers who only assist. Ask the questions only if there is someone on staff in each available group.)*

| **No.** | **Signal Functions & Essential Services** | **a. Medical doctors (GP)** | **b. Ob/Gyns** | **c. Pediatricians, neonatologists** | **d. Emergency surgical officers** | **e. Midwives** | **f. Nurses** | **g. Anesthesi-ologists, anesthetists** | **h. Health officers** |
| --- | --- | --- | --- | --- | --- | --- | --- | --- | --- |
| Q201_2 | Administer parenteral antibiotics | Yes 1  No 0 | Yes 1  No 0 | Yes 1  No 0 | Yes 1  No 0 | Yes 1  No 0 | Yes 1  No 0 | Yes 1  No 0 | Yes 1  No 0 |
| Q202_2 | Administer uterotonic drugs – parenteral oxytocics | Yes 1  No 0 | Yes 1  No 0 | Yes 1  No 0 | Yes 1  No 0 | Yes 1  No 0 | Yes 1  No 0 | Yes 1  No 0 | Yes 1  No 0 |
| Q203_2 | Administer parenteral anticonvulsants | Yes 1  No 0 | Yes 1  No 0 | Yes 1  No 0 | Yes 1  No 0 | Yes 1  No 0 | Yes 1  No 0 | Yes 1  No 0 | Yes 1  No 0 |
| Q204_2 | Perform manual removal of placenta | Yes 1  No 0 | Yes 1  No 0 | Yes 1  No 0 | Yes 1  No 0 | Yes 1  No 0 | Yes 1  No 0 | Yes 1  No 0 | Yes 1  No 0 |
| Q205_2 | Perform manual vacuum aspiration (MVA) or electric aspiration | Yes 1  No 0 | Yes 1  No 0 | Yes 1  No 0 | Yes 1  No 0 | Yes 1  No 0 | Yes 1  No 0 | Yes 1  No 0 | Yes 1  No 0 |
| Q206_2 | Perform evacuation and curettage (E&C) or D&C | Yes 1  No 0 | Yes 1  No 0 | Yes 1  No 0 | Yes 1  No 0 | Yes 1  No 0 | Yes 1  No 0 | Yes 1  No 0 | Yes 1  No 0 |
| Q207_2 | Provide medical abortion | Yes 1  No 0 | Yes 1  No 0 | Yes 1  No 0 | Yes 1  No 0 | Yes 1  No 0 | Yes 1  No 0 | Yes 1  No 0 | Yes 1  No 0 |
| Q208_2 | Perform vacuum extraction delivery | Yes 1  No 0 | Yes 1  No 0 | Yes 1  No 0 | Yes 1  No 0 | Yes 1  No 0 | Yes 1  No 0 | Yes 1  No 0 | Yes 1  No 0 |
| Q209_2 | Perform forceps delivery | Yes 1  No 0 | Yes 1  No 0 | Yes 1  No 0 | Yes 1  No 0 | Yes 1  No 0 | Yes 1  No 0 | Yes 1  No 0 | Yes 1  No 0 |
| Q210_2 | Perform obstetric surgery (e.g., cesarean delivery) | Yes 1  No 0 | Yes 1  No 0 | Yes 1  No 0 | Yes 1  No 0 | Yes 1  No 0 | Yes 1  No 0 | Yes 1  No 0 | Yes 1  No 0 |
| Q211_2 | Perform blood transfusion for mother | Yes 1  No 0 | Yes 1  No 0 | Yes 1  No 0 | Yes 1  No 0 | Yes 1  No 0 | Yes 1  No 0 | Yes 1  No 0 | Yes 1  No 0 |
| **Newborn Signal Functions** | | | | | | | | | |
| Q212_2 | Provide antenatal corticosteroids for preterm delivery | Yes 1  No 0 | Yes 1  No 0 | Yes 1  No 0 | Yes 1  No 0 | Yes 1  No 0 | Yes 1  No 0 | Yes 1  No 0 | Yes 1  No 0 |
| Q213_2 | Provide antibiotics for preterm premature/prolonged rupture of membranes | Yes 1  No 0 | Yes 1  No 0 | Yes 1  No 0 | Yes 1  No 0 | Yes 1  No 0 | Yes 1  No 0 | Yes 1  No 0 | Yes 1  No 0 |
| Q214_2 | Provide antibiotics for neonatal infections | Yes 1  No 0 | Yes 1  No 0 | Yes 1  No 0 | Yes 1  No 0 | Yes 1  No 0 | Yes 1  No 0 | Yes 1  No 0 | Yes 1  No 0 |
| Q215_2 | Administer kangaroo mother care | Yes 1  No 0 | Yes 1  No 0 | Yes 1  No 0 | Yes 1  No 0 | Yes 1  No 0 | Yes 1  No 0 | Yes 1  No 0 | Yes 1  No 0 |
| Q216_2 | Resuscitate newborn with bag and mask | Yes 1  No 0 | Yes 1  No 0 | Yes 1  No 0 | Yes 1  No 0 | Yes 1  No 0 | Yes 1  No 0 | Yes 1  No 0 | Yes 1  No 0 |
| Q217_2 | Administer oxygen to a newborn | Yes 1  No 0 | Yes 1  No 0 | Yes 1  No 0 | Yes 1  No 0 | Yes 1  No 0 | Yes 1  No 0 | Yes 1  No 0 | Yes 1  No 0 |
| Q218_2 | Administer IV fluids to a newborn | Yes 1  No 0 | Yes 1  No 0 | Yes 1  No 0 | Yes 1  No 0 | Yes 1  No 0 | Yes 1  No 0 | Yes 1  No 0 | Yes 1  No 0 |
| **Other essential services** | | | | | | | | | |
| Q219_2 | Provide focused antenatal care | Yes 1  No 0 | Yes 1  No 0 | Yes 1  No 0 | Yes 1  No 0 | Yes 1  No 0 | Yes 1  No 0 | Yes 1  No 0 | Yes 1  No 0 |
| Q220_2 | Attend normal delivery | Yes 1  No 0 | Yes 1  No 0 | Yes 1  No 0 | Yes 1  No 0 | Yes 1  No 0 | Yes 1  No 0 | Yes 1  No 0 | Yes 1  No 0 |
| Q221_2 | Fill out and use the partograph | Yes 1  No 0 | Yes 1  No 0 | Yes 1  No 0 | Yes 1  No 0 | Yes 1  No 0 | Yes 1  No 0 | Yes 1  No 0 | Yes 1  No 0 |
| Q222_2 | Provide immediate newborn care[[1]](#footnote-2) | Yes 1  No 0 | Yes 1  No 0 | Yes 1  No 0 | Yes 1  No 0 | Yes 1  No 0 | Yes 1  No 0 | Yes 1  No 0 | Yes 1  No 0 |
| Q223_2 | Provide PMTCT services | Yes 1  No 0 | Yes 1  No 0 | Yes 1  No 0 | Yes 1  No 0 | Yes 1  No 0 | Yes 1  No 0 | Yes 1  No 0 | Yes 1  No 0 |
| Q224_2 | Provide family planning counseling | Yes 1  No 0 | Yes 1  No 0 | Yes 1  No 0 | Yes 1  No 0 | Yes 1  No 0 | Yes 1  No 0 | Yes 1  No 0 | Yes 1  No 0 |
| Q225_2 | Provide temporary FP methods  (pills, condoms, injectables) | Yes 1  No 0 | Yes 1  No 0 | Yes 1  No 0 | Yes 1  No 0 | Yes 1  No 0 | Yes 1  No 0 | Yes 1  No 0 | Yes 1  No 0 |
| Q226_2 | Provide long acting reversible methods (IUD, implants) | Yes 1  No 0 | Yes 1  No 0 | Yes 1  No 0 | Yes 1  No 0 | Yes 1  No 0 | Yes 1  No 0 | Yes 1  No 0 | Yes 1  No 0 |
| Q227_2 | Provide tubal ligation | Yes 1  No 0 | Yes 1  No 0 | Yes 1  No 0 | Yes 1  No 0 | Yes 1  No 0 | Yes 1  No 0 | Yes 1  No 0 | Yes 1  No 0 |
| Q228_2 | Provide vasectomy | Yes 1  No 0 | Yes 1  No 0 | Yes 1  No 0 | Yes 1  No 0 | Yes 1  No 0 | Yes 1  No 0 | Yes 1  No 0 | Yes 1  No 0 |

SECTION 3. 24-Hour Availability

***Direct these questions to the person in charge of the maternity as we are interested in staff who work with obstetric and newborn patients. When the respondent has finished answering you can ask “Anyone else?” “Physically present” means that there is at least one staff member in this category who is physically present in the facility or nearby. “On call” means that the worker can be contacted, and if called, could reach the facility in less than 30 minutes.***

| **During a typical week, what staff are ………….?** *(do not read)* | | **Yes, mentioned** | **Not mentioned** |
| --- | --- | --- | --- |
| Q301_2 | Physically present Monday-Fri. during the day. |  |  |
|  | 1. Medical doctor (general practitioner) | 1 | 0 |
|  | 1. Obstetrician/gynecologist | 1 | 0 |
|  | 1. General surgeon | 1 | 0 |
|  | 1. Pediatrician/Neonatologist | 1 | 0 |
|  | 1. Emergency surgical officer | 1 | 0 |
|  | 1. Health officer | 1 | 0 |
|  | 1. Midwife | 1 | 0 |
|  | 1. Nurse | 1 | 0 |
|  | 1. Anesthesiologist/Anesthetist | 1 | 0 |
|  | 1. Laboratory technician | 1 | 0 |
| Q302_2 | On call Monday-Friday during the day. |  |  |
|  | 1. Medical doctor (general practitioner) | 1 | 0 |
|  | 1. Obstetrician/gynecologist | 1 | 0 |
|  | 1. General surgeon | 1 | 0 |
|  | 1. Pediatrician/Neonatologist | 1 | 0 |
|  | 1. Emergency surgical officer | 1 | 0 |
|  | 1. Health officer | 1 | 0 |
|  | 1. Midwife | 1 | 0 |
|  | 1. Nurse | 1 | 0 |
|  | 1. Anesthesiologist/Anesthetist | 1 | 0 |
|  | 1. Laboratory technician | 1 | 0 |
| Q303_2 | Physically present Monday-Friday at night. |  |  |
|  | 1. Medical doctor (general practitioner) | 1 | 0 |
|  | 1. Obstetrician/gynecologist | 1 | 0 |
|  | 1. General surgeon | 1 | 0 |
|  | 1. Pediatrician/Neonatologist | 1 | 0 |
|  | 1. Emergency surgical officer | 1 | 0 |
|  | 1. Health officer | 1 | 0 |
|  | 1. Midwife | 1 | 0 |
|  | 1. Nurse | 1 | 0 |
|  | 1. Anesthesiologist/Anesthetist | 1 | 0 |
|  | 1. Laboratory technician | 1 | 0 |
| Q304_2 | On call Monday-Friday at night. |  |  |
|  | 1. Medical doctor (general practitioner) | 1 | 0 |
|  | 1. Obstetrician/gynecologist | 1 | 0 |
|  | 1. General surgeon | 1 | 0 |
|  | 1. Pediatrician/Neonatologist | 1 | 0 |
|  | 1. Emergency surgical officer | 1 | 0 |
|  | 1. Health officer | 1 | 0 |
|  | 1. Midwife | 1 | 0 |
|  | 1. Nurse | 1 | 0 |
|  | 1. Anesthesiologist/Anesthetist | 1 | 0 |
|  | 1. Laboratory technician | 1 | 0 |
| Q305_2 | Physically present Saturday, Sunday and holidays during the day. |  |  |
|  | 1. Medical doctor (general practitioner) | 1 | 0 |
|  | 1. Obstetrician/gynecologist | 1 | 0 |
|  | 1. General surgeon | 1 | 0 |
|  | 1. Pediatrician/Neonatologist | 1 | 0 |
|  | 1. Emergency surgical officer | 1 | 0 |
|  | 1. Health officer | 1 | 0 |
|  | 1. Midwife | 1 | 0 |
|  | 1. Nurse | 1 | 0 |
|  | 1. Anesthesiologist/Anesthetist | 1 | 0 |
|  | 1. Laboratory technician | 1 | 0 |
| Q306_2 | On call Saturday, Sunday and holidays during the day. |  |  |
|  | 1. Medical doctor (general practitioner) | 1 | 0 |
|  | 1. Obstetrician/gynecologist | 1 | 0 |
|  | 1. General surgeon | 1 | 0 |
|  | 1. Pediatrician/Neonatologist | 1 | 0 |
|  | 1. Emergency surgical officer | 1 | 0 |
|  | 1. Health officer | 1 | 0 |
|  | 1. Midwife | 1 | 0 |
|  | 1. Nurse | 1 | 0 |
|  | 1. Anesthesiologist/Anesthetist | 1 | 0 |
|  | 1. Laboratory technician | 1 | 0 |
| Q307_2 | Physically present Saturday, Sunday and holidays at night. |  |  |
|  | 1. Medical doctor (general practitioner) | 1 | 0 |
|  | 1. Obstetrician/gynecologist | 1 | 0 |
|  | 1. General surgeon | 1 | 0 |
|  | 1. Pediatrician/Neonatologist | 1 | 0 |
|  | 1. Emergency surgical officer | 1 | 0 |
|  | 1. Health officer | 1 | 0 |
|  | 1. Midwife | 1 | 0 |
|  | 1. Nurse | 1 | 0 |
|  | 1. Anesthesiologist/Anesthetist | 1 | 0 |
|  | 1. Laboratory technician | 1 | 0 |
| Q308_2 | On call Saturday, Sunday and holidays at night. |  |  |
|  | 1. Medical doctor (general practitioner) | 1 | 0 |
|  | 1. Obstetrician/gynecologist | 1 | 0 |
|  | 1. General surgeon | 1 | 0 |
|  | 1. Pediatrician/Neonatologist | 1 | 0 |
|  | 1. Emergency surgical officer | 1 | 0 |
|  | 1. Health officer | 1 | 0 |
|  | 1. Midwife | 1 | 0 |
|  | 1. Nurse | 1 | 0 |
|  | 1. Anesthesiologist/Anesthetist | 1 | 0 |
|  | 1. Laboratory technician | 1 | 0 |

1. Immediate drying, skin-to-skin, delayed cord clamping, exclusive breastfeeding within the hour, etc. [↑](#footnote-ref-2)
