# Supplementary material for: Cesarean delivery rates, hospital readiness and quality of clinical management in Ethiopia: national results from two cross-sectional emergency obstetric and newborn care assessments
Source: BMC Pregnancy Childbirth. 2021 Aug 19;21:571. doi: 10.1186/s12884-021-04008-9 (PMC8377989; doi:10.1186/s12884-021-04008-9)
Supplement: Supplementary file 3 — Additional file 3: Module 3. Essential Drugs, Equipment, and Supplies. [file 12884_2021_4008_MOESM3_ESM.doc]

EmONC Assessment

MODULE 3: Essential Drugs, Equipment, and Supplies

**Interviewer ID: |___|___|___|___|**

**Date** (dd/mm/yyyy): ___ / ___ / ___

***INSTRUCTIONS:*** *This module includes five sections. You could separate the sections and ask for assistance for:*

- *Section 1 (Pharmacy) from the Pharmacist*
- *Section 2 (Labor & Delivery, Maternity) from the Head Midwife or Nurse in the Maternity*
- *Section 3 (Neonatal Care) from the Head Nurse in the Neonatal Care Unit or Maternity*
- *Section 4 (Operating Theater) from the Head Nurse in the Operating Theater*
- *Section 5 (Laboratory) from the Head Laboratory Technician*

*If the person indicated above is not available, seek someone else who can help you answer the questions for each section.*

*Not every health facility will have all of these physical spaces. Thus, please follow the skip patterns in the first questions at the beginning of each section. Sometimes you will skip sections after documenting that the facility does not have that space.*

| **Module Comments** |
| --- |
|  |

SECTION 1. Pharmacy

*These questions should be directed to the pharmacist. Most questions should be answered by circling 1 for “Yes,” or 0 for “No.” Some questions have several pre-coded answers (see Item 3 as an example). In these cases, please circle the number next to the answer given. If the respondent does not give one of the pre-coded answers, circle the number next to “Other” and specify the answer in the space provided.*

General Drugs

| **No.** | **Item** | **Response** | | **Skip to** |
| --- | --- | --- | --- | --- |
| Q101_3 | Does this facility have a pharmacy/drugstore? | Yes 1  No 0 | | If “Yes,” skip to 103_3 |
| Q102_3 | Does the facility have a supply of medicines? | Yes 1  No 0 | | If ”No,” end Section 1 & continue with Section 2 |
| Q103_3 | What is the primary source of medicines for this health facility?  *(circle multiple items if more than one is of equal importance)* | Mentioned | Not mentioned |  |
|  | 1. Government supplier | 1 | 0 |  |
|  | 1. Private pharmacy | 1 | 0 |  |
|  | 1. Non-governmental organization (NGO) / Mission | 1 | 0 |  |
|  | 1. Other *(specify)*   _________________________ | 1 | 0 |  |
| Q104_3 | Is there a drug inventory register/system?  *(this could be computerized)* | Yes 1  No 0 | | If “No,” skip to 106_3 |
| Q105_3 | *Observe drug inventory register/system or bin cards to determine if up-to-date.*  *Select 3 cards, one each from each third of the cards.* ***All*** *must be up-to-date to circle “Yes.”* | Yes 1  No 0 | |  |
| Q106_3 | When are drugs ordered?  *(circle one response)* | Order weekly/monthly/quarterly 1  Order every 6 or 12 months 2  Order whenever stocks  reach reorder level 3  Reorder when we run out 4  Never order drugs  (shipments come/kits arrive) 5  Other *(specify)* 96  ____________________________ | |  |
| Q107_3 | What is the primary source for gloves, syringes and other medical supplies?  *(circle one response)* | Government supplier 1  Private pharmacy 2  NGO / Mission 3  Other (*specify)* 96  ________________________________ | |  |
| Q108_3 | What is the most common cause of delay in the delivery of supplies or drugs?  *(circle one response)* | Inadequate transport 1  Administrative difficulties 2  Financial problems 3  Stock out at the central store 4  Other (*specify)* 96  ________________________________ | |  |
| Q109_3 | Is the pharmacy/drug store accessible 24 hours a day? | Yes 1  No 0 | |  |
| E1 | Is there adequate lighting in the pharmacy to perform your duties during the day? | Yes 1  No 0 | |  |
| E2 | Is there adequate lighting in the pharmacy to perform your duties at night? | Yes 1  No 0  No duties at night 2 | |  |
| Q110_3 | Is there a regularly used mechanism to ensure that expired drugs are not distributed? | Yes 1  No 0 | |  |
| Q111_3 | *Observe whether the “First-in First-out” system for supply management is used for gentamicin (is stock arranged by expiration date?).* | Yes 1  No 0  No gentamicin in stock 2  Did not observe 3 | |  |
| Q112_3 | *Observe whether drugs are protected from moisture, heat, or infestation (e.g., placed on shelves or slats, ventilated).* | Yes 1  No 0  Did not observe 2 | |  |
| Q113_3 | *Observe the oxytocin stock to determine if it is refrigerated* and *the temperature is monitored daily.* | Yes, refrigerated and monitored 1  Yes, refrigerated but not monitored 2  No, oxytocin not refrigerated 0  No oxytocin in stock 3  Did not observe 8 | |  |
| Q114_3 | Does this facility have at least one functioning electric/gas (liquid or compressed) refrigerator other than one used exclusively for vaccine storage (EPI)? | Yes 1  No 0 | |  |
| Q115_3 | Does this facility have at least one functioning solar refrigerator other than an EPI refrigerator? | Yes 1  No 0 | |  |

Essential Drugs

| **No.** | **Drug** | **Available**  **Yes No** | | **Skip to** |
| --- | --- | --- | --- | --- |
| Q116_3 | **Antibiotics**: Does this facility have antibiotics? | 1 | 0 | If “No” skip to Q118_3 |
| Q117_3 | Does the facility have…(*read a-r)* |  |  |  |
|  | 1. Amoxicillin (oral) | 1 | 0 |  |
|  | 1. Amoxicillin (injection) | 1 | 0 |  |
|  | 1. Ampicillin (injection) | 1 | 0 |  |
|  | 1. Augmentin | 1 | 0 |  |
|  | 1. Cephazoline sodium | 1 | 0 |  |
|  | 1. Cefixime | 1 | 0 |  |
|  | 1. Ceftriaxone | 1 | 0 |  |
|  | 1. Cefotaxime injection (for newborn) | 1 | 0 |  |
|  | 1. Clindamycin | 1 | 0 |  |
|  | 1. Cloxacillin sodium | 1 | 0 |  |
|  | 1. Erythromicin | 1 | 0 |  |
|  | 1. Oral flucloxacillin (for newborn) | 1 | 0 |  |
|  | 1. Gentamicin (injection) | 1 | 0 |  |
|  | 1. Metronidazole (injection) | 1 | 0 |  |
|  | 1. Penicillin G (Benzyl) | 1 | 0 |  |
|  | 1. Procaine benzylpenicillin (procaine penicillin G) | 1 | 0 |  |
|  | 1. Trimethoprim/sulfamethoxazole | 1 | 0 |  |
|  | 1. Tetracycline eye ointment/drops | 1 | 0 |  |
| Q118_3 | **Anticonvulsants**: Does this facility have anticonvulsants? | 1 | 0 | If “No” skip to Q120_3 |
| Q119_3 | Does the facility have…(*read a-e)* |  |  |  |
|  | 1. Magnesium sulfate (injection) 50% concentration | 1 | 0 |  |
|  | 1. Magnesium sulfate (injection) concentration other than 50% | 1 | 0 |  |
|  | 1. Diazepam (injection) | 1 | 0 |  |
|  | 1. Phenobarbital (injection) | 1 | 0 |  |
|  | 1. Phenytoin (Diphenylhydantoin) | 1 | 0 |  |
| Q120_3 | **Antihypertensives**: Does this facility have antihypertensives? | 1 | 0 | If “No” skip to Q122_3 |
| Q121_3 | Does the facility have…(*read a-d)* |  |  |  |
|  | 1. Hydralazine | 1 | 0 |  |
|  | 1. Labetalol | 1 | 0 |  |
|  | 1. Methyldopa | 1 | 0 |  |
|  | 1. Nifedipine | 1 | 0 |  |
| Q122_3 | **Oxytocics and prostaglandins**: Does this facility have oxytocics or prostaglandins? | 1 | 0 | If “No” skip to Q124_3 |
| Q123_3 | Does the facility have…(*read a-f)* |  |  |  |
|  | 1. Ergometrine | 1 | 0 |  |
|  | 1. Methylergometrine | 1 | 0 |  |
|  | 1. Misoprostol | 1 | 0 |  |
|  | 1. Oxytocin | 1 | 0 |  |
|  | 1. Prostaglandin E2 (dinoprostone) | 1 | 0 |  |
|  | 1. Prostaglandin F2 alpha | 1 | 0 |  |
| Q124_3 | **Drugs used in emergencies**: Does this facility have drugs used in emergencies? | 1 | 0 | If “No” skip to Q126_3 |
| Q125_3 | Does the facility have…*(read a-l)* |  |  |  |
|  | 1. Adrenaline (epinephrine) | 1 | 0 |  |
|  | 1. Aminophylline | 1 | 0 |  |
|  | 1. Atropine | 1 | 0 |  |
|  | 1. Calcium gluconate | 1 | 0 |  |
|  | 1. Digoxin | 1 | 0 |  |
|  | 1. Diphenhydramine | 1 | 0 |  |
|  | 1. Ephedrine | 1 | 0 |  |
|  | 1. Furosemide | 1 | 0 |  |
|  | 1. Hydrocortisone | 1 | 0 |  |
|  | 1. Naloxone | 1 | 0 |  |
|  | 1. Nitroglycerine | 1 | 0 |  |
|  | 1. Promethazine | 1 | 0 |  |
| Q126_3 | **Anesthetics**: Does this facility have anesthetics? | 1 | 0 | If “No” skip to Q128_3 |
| Q127_3 | Does the facility have…(*read a-j)* |  |  |  |
|  | 1. Halothane | 1 | 0 |  |
|  | 1. Isoflurane | 1 | 0 |  |
|  | 1. Enflurane | 1 | 0 |  |
|  | 1. Ketamine | 1 | 0 |  |
|  | 1. Lignocaine/Lidocaine 2% or 1% | 1 | 0 |  |
|  | 1. Lignocaine/lidocaine 4% | 1 | 0 |  |
|  | 1. Bupivacaine 0.5% | 1 | 0 |  |
|  | 1. Propofol | 1 | 0 |  |
|  | 1. Succinylcholine | 1 | 0 |  |
|  | 1. Pancuronium | 1 | 0 |  |
| Q128_3 | **Analgesics**: Does this facility have analgesics? | 1 | 0 | If “No” skip to Q130_3 |
| Q129_3 | Does the facility have…*(read a-h)* |  |  |  |
|  | 1. Acetylsalicylic acid | 1 | 0 |  |
|  | 1. Indomethacin | 1 | 0 |  |
|  | 1. Morphine | 1 | 0 |  |
|  | 1. Paracetamol tab | 1 | 0 |  |
|  | 1. Paracetamol suppository | 1 | 0 |  |
|  | 1. Pethidine | 1 | 0 |  |
|  | 1. Diclofenac | 1 | 0 |  |
|  | 1. Tramadol | 1 | 0 |  |
| Q130_3 | **Steroids**: Does this facility have steroids? | 1 | 0 | If “No” skip to Q132_3 |
| Q131_3 | Does the facility have…(*read a-d)* |  |  |  |
|  | 1. Betamethasone | 1 | 0 |  |
|  | 1. Dexamethasone | 1 | 0 |  |
|  | 1. Prednisone | 1 | 0 |  |
|  | 1. Prednisolone corticosteroid | 1 | 0 |  |
| Q132_3 | **IV fluids**: Does this facility have IV fluids? | 1 | 0 | If “No” skip to Q134_3 |
| Q133_3 | Does the facility have…(*read a-g)* |  |  |  |
|  | 1. Dextrose 5% with normal saline | 1 | 0 |  |
|  | 1. Dextran | 1 | 0 |  |
|  | 1. Dextrose 5% in water | 1 | 0 |  |
|  | 1. Glucose 10% | 1 | 0 |  |
|  | 1. Glucose 40% or 50% | 1 | 0 |  |
|  | 1. Normal saline | 1 | 0 |  |
|  | 1. Ringer’s lactate | 1 | 0 |  |
| Q134_3 | **Antimalarials:** Does this facility have antimalarials? | 1 | 0 | If “No” skip to 136_3 |
| Q135_3 | Does the facility have…*(read a-c)* |  |  |  |
|  | 1. Chloroquine | 1 | 0 |  |
|  | 1. Artemisium–based combination therapy (ACT) | 1 | 0 |  |
|  | 1. Quinine Dihydrochloride | 1 | 0 |  |
| Q136_3 | **Antiretrovirals** (ARVs): Does this facility have any antiretrovirals? | 1 | 0 | If “No” skip to Q138_3 |
| Q137_3 | Does the facility have…(*read a-c)* |  |  |  |
|  | 1. Post-HIV exposure prophylactic treatment | 1 | 0 |  |
|  | 1. Option B+ regimen for mother | 1 | 0 |  |
|  | 1. Option B+ regimen for newborn | 1 | 0 |  |
| Q138_3 | **Contraceptives**: Does this facility have any contraceptives? | 1 | 0 | If “No” 🡪 skip to Q140_3 |
| Q139_3 | Does the facility have…(*read a-h)* |  |  |  |
|  | 1. Combined oral contraceptives | 1 | 0 |  |
|  | 1. Progestin only pills | 1 | 0 |  |
|  | 1. Implants | 1 | 0 |  |
|  | 1. 3-month injectables | 1 | 0 |  |
|  | 1. Intrauterine devices (IUDs) | 1 | 0 |  |
|  | 1. Male condoms | 1 | 0 |  |
|  | 1. Female condoms | 1 | 0 |  |
|  | 1. Emergency contraception | 1 | 0 |  |

| Q140_3 | **Other drugs and supplies**: Does this facility have … *(read a-q)* |  |  |  |
| --- | --- | --- | --- | --- |
|  | 1. Vitamin K (for newborn) | 1 | 0 |  |
|  | 1. Chlorhexidine (4% gel for cord cleansing) | 1 | 0 |  |
|  | 1. Nystatin (oral) (for newborn) | 1 | 0 |  |
|  | 1. Oral rehydration solution | 1 | 0 |  |
|  | 1. Gentian violet paint | 1 | 0 |  |
|  | 1. Ferrous sulfate or fumarate | 1 | 0 |  |
|  | 1. Folic acid | 1 | 0 |  |
|  | 1. Ferrous sulfate together with folic acid | 1 | 0 |  |
|  | 1. Heparin | 1 | 0 |  |
|  | 1. Magnesium trisilicate | 1 | 0 |  |
|  | 1. Polio 0 | 1 | 0 |  |
|  | 1. BCG vaccine | 1 | 0 |  |
|  | 1. Anti-tetanus serum / TAT | 1 | 0 |  |
|  | 1. Tetanus toxoid vaccine | 1 | 0 |  |
|  | 1. Anti-Rho (D) immune globulin | 1 | 0 |  |
|  | 1. Insecticide-treated bednets (ITN) | 1 | 0 |  |
|  | 1. Mebendazole | 1 | 0 |  |
|  | 1. Metoclopramide | 1 | 0 |  |

Stock Outs

| **No.** | **Item** | **Response** | | **Skip** |
| --- | --- | --- | --- | --- |
| Q141_3 | Have you had a stock out of any of the following items in the last 3 months? *(read items)*   1. Gentamicin (injection) 2. Magnesium sulfate 3. Ketamine 4. Dexamethasone 5. Antiretrovirals (ARV) 6. Oxytocin | Yes  1  1  1  1  1  1 | No  0  0  0  0  0  0 | If the answer to f. Oxytocin is “No, skip to Q142_3 |
| E3 | Did the stock out of oxytocin occur because there was damage to the supply from an interruption in the cold chain? | 1 | 0 |  |
| Q142_3_333 | In the last 12 months, has there been an interruption in the safe oxygen supply (cylinder or concentrator) …? | Facility never has O_2_ | Has O_2_ and supply interrupted | Has O_2_ with no interruption |
|  | 1. In labor and delivery | 9 | 1 | 0 |
|  | 1. In the neonatal ward | 9 | 1 | 0 |
|  | 1. In the pediatric ward | 9 | 1 | 0 |
| Q143_33 | If there was an interruption in safe oxygen supply in any area of the facility, was the interruption ever due to lack of electricity? | Yes 1  No 0  Never had O_2_ 8  No interruptions 9 | |  |

| Section 1 Comments |
| --- |
|  |

INS_201_3 SECTION 2. Labor and Delivery and Maternity

*Direct these questions to the Head Midwife or Nurse in the maternity. Most questions should be answered by circling 1 for “Yes” or 0 for “No.”*

2A. Infection Prevention

| **No.** | **Item** | **Is at least 1 available and functional?**  **Yes No** | |
| --- | --- | --- | --- |
| Q201_3 | Infection Prevention Basic Items: Does this facility have…  *(read a-q)* |  |  |
|  | 1. Soap | 1 | 0 |
|  | 1. Antiseptics | 1 | 0 |
|  | 1. Disposable latex examination gloves | 1 | 0 |
|  | 1. Elbow length gloves | 1 | 0 |
|  | 1. Heavy duty gloves | 1 | 0 |
|  | 1. Eye shields | 1 | 0 |
|  | 1. Mask | 1 | 0 |
|  | 1. Non-sterile protective clothing | 1 | 0 |
|  | 1. Decontamination container | 1 | 0 |
|  | 1. Bleach or bleaching powder (chlorine) | 1 | 0 |
|  | 1. Prepared disinfection solution | 1 | 0 |
|  | 1. Regular trash bin | 1 | 0 |
|  | 1. Covered contaminated waste trash bin | 1 | 0 |
|  | 1. Puncture-proof sharps container | 1 | 0 |
|  | 1. Mayo stand/table (or equivalent to establish sterile field) | 1 | 0 |
|  | 1. Surgeon’s hand brush with nylon bristles | 1 | 0 |
|  | 1. Surgeon’s boots | 1 | 0 |
| Q202_3 | Disinfectants and antiseptics: Does the facility have*…(read a-f)* |  |  |
|  | 1. Chlorhexidine (4%) gel | 1 | 0 |
|  | 1. Chlorhexidine solution (savalon) | 1 | 0 |
|  | 1. Ethanol 75% | 1 | 0 |
|  | 1. Ethanol 95% | 1 | 0 |
|  | 1. Polyvidone iodine | 1 | 0 |
|  | 1. Alcohol-based rub | 1 | 0 |

**2B. Infrastructure**

| **No.** | **Item** | **Is at least 1 available and functional?** | |
| --- | --- | --- | --- |
| Q203_3 | Infrastructure: Does the facility have*…(read a-i)* |  |  |
|  | 1. Sufficient light source to perform tasks during the day | 1 | 0 |
|  | 1. Sufficient light source to perform tasks at night | 1 | 0 |
|  | 1. Means of ventilation | 1 | 0 |
|  | 1. Functioning toilet for patient use | 1 | 0 |
|  | 1. Heating/heating arrangements | 1 | 0 |
|  | 1. Functional fan/air conditioning | 1 | 0 |
|  | 1. Curtains/means of providing patient privacy | 1 | 0 |
|  | 1. Waiting area for visitors and family | 1 | 0 |
|  | 1. Functioning toilet for visitors’ and family use | 1 | 0 |
| Q204_3 | In the last 3 months, have any inpatients shared beds at any time before or after delivery? | 1 | 0 |
| Q205_3 | In the last 3 months, have delivery patients slept on the floor? | 1 | 0 |
| Q206_3 | In the last 3 months, have delivery patients delivered on the floor, in a corridor or bathroom? | 1 | 0 |

2C. Guidelines and Protocols

| **No.** | Item | **Response**  **Yes No** | |
| --- | --- | --- | --- |
| Q207_3 | Are there guidelines, protocols or job aids available for*…(read a-l)* |  |  |
|  | 1. Safe abortion technical guideline | 1 | 0 |
|  | 1. Prevention of mother-to-child transmission of HIV | 1 | 0 |
|  | 1. Management protocol on selected obstetric topics (for health centers, for hospitals) | 1 | 0 |
|  | 1. Infection prevention guideline | 1 | 0 |
|  | 1. Reimbursement protocol | 1 | 0 |
|  | 1. Integrated management of pregnancy, childbirth, postpartum and newborn care (focus on routine care) |  | 0 |
|  | 1. Care for preterm or low birth weight babies, including kangaroo mother care | 1 | 0 |
|  | 1. Neonatal resuscitation | 1 | 0 |
|  | 1. Treatment of infections in young infants (IMNCI) | 1 | 0 |
|  | 1. Referral and counter-referral | 1 | 0 |
|  | 1. Infection prevention for HIV/AIDS (universal precautions) | 1 | 0 |
|  | 1. Family planning | 1 | 0 |

2D. Equipment and Supplies

| **No.** | **Item** | | **Is at least 1 available and functional?**  **Yes No** | | |
| --- | --- | --- | --- | --- | --- |
| General: Does the facility have*…(read all items below)* | | | | | |
| Q208_3 | Filled oxygen cylinder with cylinder carrier and key to open valve | 1 | | 0 | |
| Q209_3 | Ultrasound | 1 | | 0 | |
| Q210_3 | CTG (cardiotocography) external or internal | 1 | | 0 | |
| Q211_3 | Blood pressure cuff | 1 | | 0 | |
| Q212_3 | Stethoscope (for adults) | 1 | | 0 | |
| Q213_3 | Fetal stethoscope | 1 | | 0 | |
| Q214_3 | Doppler | 1 | | 0 | |
| Q215_3 | Kidney basins | 1 | | 0 | |
| Q216_3 | Sponge bowls | 1 | | 0 | |
| Q217_3 | Clinical thermometer | 1 | | 0 | |
| Q218_3 | Low reading thermometer (32 or 35 degree C) | 1 | | 0 | |
| Q219_3 | Scissors | 1 | | 0 | |
| Q220_3 | Needles and syringes (10-20cc) | 1 | | 0 | |
| Q221_3 | Syringes (e.g. 1ml, 2ml, 5ml, 10ml, 15ml, etc.) | 1 | | 0 | |
| Q222_3 | Needles (23-25 gauge) | 1 | | 0 | |
| Q223_3 | Suture needles/suture materials | 1 | | 0 | |
| Q224_3 | Catheter for IV line/adult cannulae (16-18) | 1 | | 0 | |
| Q225_3 | IV Infusion stand(s) | 1 | | 0 | |
| Q226_3 | Urinary catheters | 1 | | 0 | |
| Q227_3 | IV cannula 24 gauge | 1 | | 0 | |
| Q228_3 | Dipstick for urinalysis (protein, sugar, bacteriuria, bilirubin, etc.) | 1 | | 0 | |
| Q229_3 | Adult ventilator bag and mask | 1 | | 0 | |
| Q230_3 | Wheelchair | 1 | | 0 | |
| Q231_3 | Stretcher with trolley | 1 | | 0 | |
| Q232_3 | Examination table | 1 | | 0 | |
| Q233_3 | Labor/delivery table with stirrups | 1 | | 0 | |
| Q234_3 | Labor/delivery table without stirrups | 1 | | 0 | |
| Q235_3 | Dressing forceps | 1 | | 0 | |
| Q236_3 | Partograph form | 1 | | 0 | |
| Q237_3 | Watch or clock with second hand that can be easily seen | 1 | | 0 | |
| Q238_3 | Measuring tape | 1 | | 0 | |
| Q239_3 | Obstetric wheel (for measuring gestational age) | 1 | | 0 | |
| Q240_3 | Tubing for oxygen administration | 1 | | 0 | |
| Q241_3 | Pulse oximeter | 1 | | 0 | |
| Q242_3 | Apnea monitor | 1 | | 0 | |
| Q243_3 | Instrument trolley | 1 | | 0 | |
| Q244_3 | Instrument tray | 1 | | 0 | |
| Q245_3 | Beds | 1 | | 0 | |
| Q246_3 | Linens | 1 | | 0 | |
| Q247_3 | Blankets for cold weather | 1 | | 0 | |
| Q248_3 | Water filter (or other means to make potable water available to patients and staff) | 1 | | 0 | |
| Q249_3 | HIV rapid testing kit | 1 | | 0 | |
| Q250_3 | Delivery set/pack: Does the facility have*…(read a-g)* |  | |  | |
|  | 1. Complete delivery sets | 1 | | 0 | |
|  | 1. How many complete delivery sets are there in total?   *(write number)* | \|____\|____\|____\| | | | |
|  | 1. Disposable latex gloves (short) | 1 | | 0 | |
|  | 1. Long gloves | 1 | | 0 | |
|  | 1. Plastic sheeting | 1 | | 0 | |
|  | 1. Gauze swabs | 1 | | 0 | |
|  | 1. Cloths or towels for drying the baby | 1 | | 0 | |
| Q251_3 | Does the facility have *…(read a-d)* |  | |  | |
|  | 1. A complete episiotomy/perineal/ set? | 1 | | 0 | |
|  | 1. How many complete episiotomy/perineal/ set are there?   *(write number)* | \|____\|____\|____\| | | | |
|  | 1. Cervical exploration and repair set? | 1 | | 0 | |
|  | 1. How many complete cervical exploration and repair set are there? *(write number)* | \|____\|____\|____\| | | | |
| Q252_3 | Instrumental vaginal delivery sets: Does the facility have…  *(read a-d)* |  | | |  |
|  | 1. Functional vacuum extractor with different size cups | 1 | | | 0 |
|  | 1. Obstetric forceps, outlet | 1 | | | 0 |
|  | 1. Obstetric forceps, low | 1 | | | 0 |
|  | 1. Obstetric forceps, breech | 1 | | | 0 |
| Q253_3 | Uterine evacuation**:** Does the facility have…*(read a-h)* |  | | |  |
|  | 1. Electric vacuum aspiration machine | 1 | | | 0 |
|  | 1. Vaginal speculum, Sims | 1 | | | 0 |
|  | 1. Sponge (ring) forceps | 1 | | | 0 |
|  | 1. Postpartum curette | 1 | | | 0 |
|  | 1. Uterine dilators, sizes 13-27 | 1 | | | 0 |
|  | 1. Sharp uterine curettes, size 0 or 00 | 1 | | | 0 |
|  | 1. Blunt uterine curettes, size 0 or 00 | 1 | | | 0 |
|  | 1. Uterine sound | 1 | | | 0 |
| Q254_3 | Manual vacuum aspiration: Does the facility have… *(read a-f)* |  | | |  |
|  | 1. Complete manual vacuum aspiration set | 1 | | | 0 |
|  | 1. Vacuum aspirators/syringes | 1 | | | 0 |
|  | 1. Silicone lubricant (for lubricating O-ring) | 1 | | | 0 |
|  | 1. Other oil (for lubricating O-ring) | 1 | | | 0 |
|  | 1. Flexible cannulae, 4-6mm | 1 | | | 0 |
|  | 1. Flexible cannulae, 7-12mm | 1 | | | 0 |
| Q255_3 | Dressing instrument set: Does the facility have…*(read a-i)* (S/S=stainless steel) |  | | |  |
|  | 1. Gallipot bowl or jar S/S | 1 | | | 0 |
|  | 1. Dissecting forceps 1x2 teeth 140mm | 1 | | | 0 |
|  | 1. Needle holder 180mm S/S | 1 | | | 0 |
|  | 1. Scissors sharp straight 120mm S/S | 1 | | | 0 |
|  | 1. Scissors flat curved 180mm S/S | 1 | | | 0 |
|  | 1. Sponge (ring) forceps | 1 | | | 0 |
|  | 1. Artery forceps straight/mosquito 130mm S/S | 1 | | | 0 |
|  | 1. Other type of scissors | 1 | | | 0 |
|  | 1. Other type of artery forceps | 1 | | | 0 |
| Q256_3 | Gynecological equipment: Does the facility have…*(read a-f)* (S/S=stainless steel) |  | |  | |
|  | 1. Vaginal speculum, Sims | 1 | | 0 | |
|  | 1. Vaginal speculum, Cusco, virgin size, 75x17mm | 1 | | 0 | |
|  | 1. Vaginal speculum, Cusco, adult sized | 1 | | 0 | |
|  | 1. Uterine sound, graduated, 305mm, S/S | 1 | | 0 | |
|  | 1. Tenaculum single tooth/multi teeth | 1 | | 0 | |
|  | 1. Scissors straight sharp 145mm S/S | 1 | | 0 | |
| Q257_3 | Craniotomy equipment: Does the facility have…read (a-d) (S/S=stainless steel) |  | |  | |
|  | 1. Decapitation hook S/S | 1 | | 0 | |
|  | 1. Craniotomy forceps S/S | 1 | | 0 | |
|  | 1. Embryotomy scissors | 1 | | 0 | |
|  | 1. Perforator | 1 | | 0 | |

2E. Autoclave Room

| **No.** | Item | **Is at least 1 available and functional?**  **Yes No** | |
| --- | --- | --- | --- |
| Q258_3 | Does the maternity have its own sterilization equipment and space or does it use a centralized area shared by other services?  Has its own equipment and space 1  Shares equipment and space with other services 2  Other *(specify)* _________________________________ 96 | | |
| Q259_3 | Sterilization area items: Does the area have…*(read a-g)* |  |  |
|  | 1. Autoclave with temperature and pressure gauges | 1 | 0 |
|  | 1. Hot air sterilizer (dry oven) | 1 | 0 |
|  | 1. Steam sterilizer | 1 | 0 |
|  | 1. Steam instrument sterilizer/pressure cooker, electric | 1 | 0 |
|  | 1. Sterilizer/pressure cooker, kerosene heated | 1 | 0 |
|  | 1. Sterilization drum | 1 | 0 |
|  | 1. Sterilization drum stand | 1 | 0 |

2F. Miscellaneous

| **No.** | **Item** | **Is at least 1 available and functional?**  **Yes No** | |
| --- | --- | --- | --- |
| Q260_3 | Does the facility have a functioning incinerator? | 1 | 0 |
| Q261_3 | Does the facility have a placental pit? | 1 | 0 |
| Q262_3 | Is food provided to patients by the facility? | 1 | 0 |
| Q263_3 | Are there empty beds for the next patients? |  |  |
|  | 1. First stage (labor) | 1 | 0 |
|  | 1. Postnatal | 1 | 0 |
|  | 1. Obstetrics | 1 | 0 |
| Q264_3 | *For observation only*: Can you see any liquid spills or trash on the floor? | 1 | 0 |

| **Section 2 Comments** |
| --- |
|  |

INS_301_3 SECTION 3. Neonatal Care

*Direct these questions to the Head Nurse in charge of neonatal care. If there is no Head Nurse, direct these questions to the nurse in-charge of neonatal care at the time of your visit. All questions should be answered by circling 1 for “Yes,” or 0 for “No.” Neonatal care may take place in a designated physical space as part of the delivery room or postnatal ward or it may be delivered in a separate area. This space is often called a newborn corner or neonatal care unit.*

3A. Equipment and Supplies

| **No.** | **Item** | **Is at least 1 available and functional?**  **Yes No** | |
| --- | --- | --- | --- |
| Q301_3 | Material for the newborn: Does the facility have…*(read a-e)* |  |  |
|  | 1. Baby weighing scale | 1 | 0 |
|  | 1. Cord ties / clips | 1 | 0 |
|  | 1. Thermometer for newborn | 1 | 0 |
|  | 1. Caps or hats to prevent heat loss | 1 | 0 |
|  | 1. Towels / blanket or cloth for newborn | 1 | 0 |
| Q302_3 | Neonatal resuscitation pack: Does the facility have…*(read a-l)* |  |  |
|  | 1. Newborn resuscitation table | 1 | 0 |
|  | 1. Mucus extractor/simple suction | 1 | 0 |
|  | 1. Neonatal face mask, size 0 | 1 | 0 |
|  | 1. Neonatal face mask, size 1 | 1 | 0 |
|  | 1. Neonatal size ambu (ventilatory) bag | 1 | 0 |
|  | 1. Suction catheter, 10, 12 Ch | 1 | 0 |
|  | 1. Infant laryngoscope with spare bulb and batteries | 1 | 0 |
|  | 1. Endotracheal tubes, 3.5mm, 3.0mm, 2.5mm | 1 | 0 |
|  | 1. Disposable uncuffed tracheal tubes, sizes 2.0 to 3.5 | 1 | 0 |
|  | 1. Suction aspirator (operated by foot or electrically) | 1 | 0 |
|  | 1. Mucus trap for suction | 1 | 0 |
|  | 1. Newborn anatomical model (for practice) | 1 | 0 |
| Q303_3 | Is the equipment for resuscitation within the delivery unit always accessible? | 1 | 0 |
| Q304_3 | Are there decontamination supplies for bag and mask? | 1 | 0 |
| Q305_3 | Small or sick newborn care: Does the facility have…*(read a-t)* |  |  |
|  | 1. Register for sick babies | 1 | 0 |
|  | 1. Daily patient chart | 1 | 0 |
|  | 1. IV fluid (neonatal giving) set | 1 | 0 |
|  | 1. Exchange transfusion set | 1 | 0 |
|  | 1. Umbilical catheter | 1 | 0 |
|  | 1. Syringes (0.5, 1.0 ml) | 1 | 0 |
|  | 1. Radiant warmer | 1 | 0 |
|  | 1. Incubator | 1 | 0 |
|  | 1. Designated space or beds for kangaroo mother care | 1 | 0 |
|  | 1. KMC register | 1 | 0 |
|  | 1. Nasogastric feeding tube # 4 | 1 | 0 |
|  | 1. Cup and spoon for infant feeding | 1 | 0 |
|  | 1. Cup for breast milk expression | 1 | 0 |
|  | 1. Icterometer | 1 | 0 |
|  | 1. Fluorescent tubes for phototherapy to treat jaundice | 1 | 0 |
|  | 1. Oxygen source | 1 | 0 |
|  | 1. Laryngoscope newborn size | 1 | 0 |
|  | 1. Respirator for neonates | 1 | 0 |
|  | 1. CPAP (continuous positive airway pressure) machine | 1 | 0 |
|  | 1. 4% Chlorhexidine gel | 1 | 0 |

| Section 3 Comments |
| --- |
|  |

INS_401_3 SECTION 4. Operating Theater

*Direct these questions to the Head Nurse in the OT. All questions should be answered by circling 1 for “Yes,” or 0 for “No.”*

4A. General

| **No.** | **Item** | **Response** |
| --- | --- | --- |
| Q401_3 | Does this facility have an operating theater for major operations, including cesarean delivery? | Yes 1  No 0  If “No” 🡪 skip to Section 5. |
| Q402_3 | Is there one or more separate operating theaters only for obstetric patients? | Yes 1  No 0 |

4B. Equipment and Supplies

| **No.** | **Item** | **Is at least 1 available and functional?**  **Yes No** | |
| --- | --- | --- | --- |
| Q403_3 | Basic items: Does the facility have…*(read all)* |  |  |
|  | 1. Operating table | 1 | 0 |
|  | 1. Light, adjustable, shadow less / overhead | 1 | 0 |
|  | 1. Surgical drapes | 1 | 0 |
|  | 1. Syringes, 5ml | 1 | 0 |
|  | 1. Syringes, 10ml | 1 | 0 |
|  | 1. Syringes, 20ml | 1 | 0 |
|  | 1. Needles, 21, 22, 23 | 1 | 0 |
|  | 1. Adequate light to provide surgery during the day | 1 | 0 |
|  | 1. Adequate light to provide surgery at night | 1 | 0 |
| Q404_3 | Obstetric laparotomy/cesarean delivery pack/mini-lap: Does the facility have…*(read all)* |  |  |
|  | 1. Stainless steel instrument tray with cover | 1 | 0 |
|  | 1. Towel clips | 1 | 0 |
|  | 1. Sponge (ring) forceps, 22.5cm | 1 | 0 |
|  | 1. Straight artery forceps, 16cm | 1 | 0 |
|  | 1. Uterine hemostasis forceps, 20cm | 1 | 0 |
|  | 1. Needle holder | 1 | 0 |
|  | 1. Surgical knife handle, No. 3 | 1 | 0 |
|  | 1. Surgical knife handle, No. 4 | 1 | 0 |
|  | 1. Surgical knife blades | 1 | 0 |
|  | 1. Triangular point suture needles, 7.3cm/size 6 | 1 | 0 |
|  | 1. Round-bodied needles, No. 12/size 6 | 1 | 0 |
|  | 1. Abdominal retractor, size 3 | 1 | 0 |
|  | 1. Abdominal retractors, double-ended | 1 | 0 |
|  | 1. Operating scissors, curved, blunt 17cm | 1 | 0 |
|  | 1. Operating scissors, straight, blunt 17cm | 1 | 0 |
|  | 1. Scissors, straight, 23cm | 1 | 0 |
|  | 1. Suction nozzle | 1 | 0 |
|  | 1. Suction tube, 22.5cm, 23 French gauge | 1 | 0 |
|  | 1. Intestinal clamps, curved, 22.5cm | 1 | 0 |
|  | 1. Intestinal clamps, straight, 22.5cm | 1 | 0 |
|  | 1. Dressing (tissue) forceps, non-toothed, 15cm | 1 | 0 |
|  | 1. Dressing (tissue) forceps, non-toothed, 25cm | 1 | 0 |
|  | 1. Sutures (different sizes and types) | 1 | 0 |
|  | 1. Mini-laparotomy kit (for female sterilization) | 1 | 0 |
| Q405_3 | Anesthesia equipment: Does the facility have…*(read all)* |  |  |
|  | 1. Anesthetic face masks | 1 | 0 |
|  | 1. Oropharyngeal airways | 1 | 0 |
|  | 1. Laryngoscopes with spare bulbs and batteries | 1 | 0 |
|  | 1. Endotracheal tubes with cuffs, 8mm | 1 | 0 |
|  | 1. Endotracheal tubes with cuffs, 10mm | 1 | 0 |
|  | 1. Endotracheal tubes 2.5 mm – 3.5 mm | 1 | 0 |
|  | 1. Intubating forceps | 1 | 0 |
|  | 1. Endotracheal tube connectors, plastic, 15 mm (connect directly to breathing valve; three for each tube size) | 1 | 0 |
|  | 1. Spinal needles, 18 gauge to 25 gauge | 1 | 0 |
|  | 1. Suction aspirator, foot-operated | 1 | 0 |
|  | 1. Suction aspirator, electric | 1 | 0 |
|  | 1. Anesthetic vaporizers (draw-over system) | 1 | 0 |
|  | 1. Oxygen cylinders with manometer and flowmeter (low flow) tubes and connectors | 1 | 0 |

| Section 4 Comments |
| --- |
|  |

INS_501_3 SECTION 5. Laboratory and Blood Bank

Direct questions to the Head Technician. All of the questions should be answered by circling 1 for “Yes,” or 0 for “No.”

5A. General

| **No.** | **Item** | **Available** |
| --- | --- | --- |
| Q501_3 | Does this facility have a laboratory? | Yes 1  No…….... 0  *If “No” 🡪 skip to 506_3.* |
| Q502_3 | Does the laboratory have a written set of guidelines? | Yes 1  No 0 |

5B. Equipment and Supplies

| **No.** | **Item** | **Is at least 1 available and functional?**  **Yes No** | |
| --- | --- | --- | --- |
| Q503_3 | Provision of donor blood for transfusion: Does the facility have…(*read a-p*) |  |  |
|  | 1. Refrigerator for blood bank | 1 | 0 |
|  | 1. Test tubes, various sizes | 1 | 0 |
|  | 1. Microscope slides | 1 | 0 |
|  | 1. Compound microscope for cross-matching | 1 | 0 |
|  | 1. Microscope illuminator | 1 | 0 |
|  | 1. Blood lancets | 1 | 0 |
|  | 1. Cotton wool | 1 | 0 |
|  | 1. Rack | 1 | 0 |
|  | 1. 8.5g/l Sodium chloride solution | 1 | 0 |
|  | 1. 20% Bovine albumin | 1 | 0 |
|  | 1. Centrifuge, electric | 1 | 0 |
|  | 1. Centrifuge, hand driven | 1 | 0 |
|  | 1. 37^o^ C Water bath (or incubator) | 1 | 0 |
|  | 1. Pipettes volumetric, various sizes | 1 | 0 |
|  | 1. Blood typing and cross-matching reagents | 1 | 0 |
|  | 1. Bags for collecting blood | 1 | 0 |
| Q504_3 | Blood collection and screening tests: Does the facility have…(*read a-k*) |  |  |
|  | 1. Airway needle for giving blood | 1 | 0 |
|  | 1. Artery forceps | 1 | 0 |
|  | 1. Anticoagulant bottles | 1 | 0 |
|  | 1. Scale for blood collection | 1 | 0 |
|  | 1. Hepatitis B test | 1 | 0 |
|  | 1. Hepatitis C test | 1 | 0 |
|  | 1. HIV Rapid Diagnostic Test (RDT) kit | 1 | 0 |
|  | 1. Syphilis test | 1 | 0 |
|  | 1. TB microscopy (slides, stain) | 1 | 0 |
|  | 1. Malaria RDT kit | 1 | 0 |
|  | 1. Pregnancy test | 1 | 0 |
| Q505_3 | Laboratory supplies: Does the facility have…(*read a-ll)* |  |  |
|  | 1. Microscope | 1 | 0 |
|  | 1. Immersion oil | 1 | 0 |
|  | 1. Glass rods | 1 | 0 |
|  | 1. Sink or staining tank | 1 | 0 |
|  | 1. Measuring cylinder, polypropylene, various sizes | 1 | 0 |
|  | 1. Wash bottle | 1 | 0 |
|  | 1. Bottle with buffered water | 1 | 0 |
|  | 1. Timer clock with alarm | 1 | 0 |
|  | 1. Rack for drying slides | 1 | 0 |
|  | 1. Giemsa stain | 1 | 0 |
|  | 1. Wright stain | 1 | 0 |
|  | 1. May Grünwald stain | 1 | 0 |
|  | 1. Funnel and filter paper | 1 | 0 |
|  | 1. Methanol | 1 | 0 |
|  | 1. Refrigerator for laboratory supplies | 1 | 0 |
|  | 1. Glass containers | 1 | 0 |
|  | 1. Counting chamber (differential counter) | 1 | 0 |
|  | 1. Pipette, 5ml | 1 | 0 |
|  | 1. Pipette, graduated 1.0ml | 1 | 0 |
|  | 1. Dropping pipette | 1 | 0 |
|  | 1. Cover slips | 1 | 0 |
|  | 1. Petri dishes | 1 | 0 |
|  | 1. Bowls, kidney dishes, various sizes, S/S | 1 | 0 |
|  | 1. Turk diluting solution | 1 | 0 |
|  | 1. Tally counter | 1 | 0 |
|  | 1. Hemoglobinometer and hydrochloric acid solution | 1 | 0 |
|  | 1. Spectrophotometer (symex, screenplus) | 1 | 0 |
|  | 1. Microhematocrit centrifuge (manual or electric) | 1 | 0 |
|  | 1. Balance for reading results | 1 | 0 |
|  | 1. Heparinized capillary tubes, 75mm x 1.5mm | 1 | 0 |
|  | 1. Spirit lamp | 1 | 0 |
|  | 1. Ethanol | 1 | 0 |
|  | 1. Test tubes | 1 | 0 |
|  | 1. Test tube rack | 1 | 0 |
|  | 1. Beakers, various sizes | 1 | 0 |
|  | 1. Ammonia | 1 | 0 |
|  | 1. Lugol’s iodine solution | 1 | 0 |
|  | 1. CD4 machine | 1 | 0 |

5C. Blood Transfusion Supply Stock

| **No.** | **Item** | **Response** |
| --- | --- | --- |
| Q506_3 | How many units of blood ready for transfusion do you have in stock? | |___|___|___| |

| **Section 5 Comments** |
| --- |
|  |
