# Supplementary material for: Cesarean delivery rates, hospital readiness and quality of clinical management in Ethiopia: national results from two cross-sectional emergency obstetric and newborn care assessments
Source: BMC Pregnancy Childbirth. 2021 Aug 19;21:571. doi: 10.1186/s12884-021-04008-9 (PMC8377989; doi:10.1186/s12884-021-04008-9)
Supplement: Supplementary file 4 — Additional file 4: Module 4. Facility Case Summary. [file 12884_2021_4008_MOESM4_ESM.doc]

EmONC Assessment

MODULE 4: Facility Case Summary

Interviewer Name

**Date** (dd/mm/yyyy): ___ / ___ / ___

*INSTRUCTIONS: Please begin this module by telling the medical director or matron that you want information on the number of deliveries, the mode of delivery, obstetric and abortion-related complications, newborn morbidities and the number of maternal and newborn deaths that have occurred in the past year. S/he should be able to tell you which registers and data sources are in use at this facility that might be helpful. Refer to the Flow Chart on page 38 of the Data Collector’s Manual and use it as a reminder of which registers you should consult for which items. These will be the data sources you will consult for this module.*

SECTION 1. Registers

***INS100_4:*** *Ask whether the registers below are used in this facility for maternal and newborn care and if there are additional registers not listed here.*

| **No.** | **Registers and data sources** | **Is it used at this facility?** | |
| --- | --- | --- | --- |
|
|  | | **Yes** | **No** |
| Q101_4 | Labor and delivery ward register | 1 | 0 |
| Q102_4 | Postpartum ward register | 1 | 0 |
| Q103_4 | Newborn unit register | 1 | 0 |
| Q104_4 | Operating theater register | 1 | 0 |
| Q105_4 | Gynecology ward / Inpatient department register | 1 | 0 |
| Q106_4 | Safe abortion / postabortion (PAC) register | 1 | 0 |
| Q107_4 | Discharge register | 1 | 0 |
| Q108_4 | Death / mortuary register | 1 | 0 |
| Q109_4 | PMTCT of HIV register | 1 | 0 |
| Q110_4 | Referral / counter referral register | 1 | 0 |
| Q111_4 | Antenatal care register | 1 | 0 |
| Q112_4 | Maternal death reporting format / pad | 1 | 0 |
| Q113_4 | Family planning register | 1 | 0 |
| Q114a_4 | Other *(specify)* | 1 | 0 |
| Q114b_4 | Other (*specify)* | 1 | 0 |

SECTION 2: Data for Indicators

***INS200_4:*** *Provide the number of cases for each category. The months refer to the Gregorian calendar year 2015.*

| **No.** | | **Item** | | **Jan** | | **Feb** | | **Mar** | | **Apr** | | **May** | | **June** | | **July** | | **Aug** | | **Sept** | | **Oct** | | | **Nov** | | **Dec** |
| --- | --- | --- | --- | --- | --- | --- | --- | --- | --- | --- | --- | --- | --- | --- | --- | --- | --- | --- | --- | --- | --- | --- | --- | --- | --- | --- | --- |
| **Deliveries** | | | | | | | | | | | | | | | | | | | | | | | | | | | |
| Q201_4 | | Spontaneous vaginal deliveries (normal,  breech, face) | |  | |  | |  | |  | |  | |  | |  | |  | |  | |  | | |  | |  |
| Q202_4 | | Deliveries with vacuum extraction or forceps | |  | |  | |  | |  | |  | |  | |  | |  | |  | |  | | |  | |  |
| Q203_4 | | Craniotomies/embryotomies/destructive deliveries | |  | |  | |  | |  | |  | |  | |  | |  | |  | |  | | |  | |  |
| Q204_4 | | Cesarean deliveries (emergencies and electives) | |  | |  | |  | |  | |  | |  | |  | |  | |  | |  | | |  | |  |
| Q205_4 | | Laparotomies for ruptured uterus | |  | |  | |  | |  | |  | |  | |  | |  | |  | |  | | |  | |  |
| Of the total deliveries, how many mothers are aged < 18 years | | | | | | | | | | | | | | | | | | | | | | | | |  | |  |
| Q206_4 | | Mothers < 18 years of age | |  | |  | |  | |  | |  | |  | |  | |  | |  | |  | | |  | |  |
| **Postabortion Care (PAC) and Family Planning** | | | | | | | | | | | | | | | | | | | | | | | | | | | |
| Q207_4 | | PAC cases (no severe complications) | |  | |  | |  | |  | |  | |  | |  | |  | |  | |  | | |  | |  |
| Q208_4 | | Safe abortion cases | |  | |  | |  | |  | |  | |  | |  | |  | |  | |  | | |  | |  |
| Q209_4 | | Postabortion women discharged with a family planning method | |  | |  | |  | |  | |  | |  | |  | |  | |  | |  | | |  | |  |
| Q210_4 | | Postpartum women discharged with a family planning method | |  | |  | |  | |  | |  | |  | |  | |  | |  | |  | | |  | |  |
| **Direct Obstetric Complications** | | | | | | | | | | | | | | | | | | | | | | | | | | | |
| Q211_4 | | Antepartum hemorrhage | |  | |  | |  | |  | |  | |  | |  | |  | |  | |  | | |  | |  |
| Q212_4 | | Postpartum hemorrhage | |  | |  | |  | |  | |  | |  | |  | |  | |  | |  | | |  | |  |
| Q213_4 | | Retained placenta | |  | |  | |  | |  | |  | |  | |  | |  | |  | |  | | |  | |  |
| Q214_4 | | Prolonged/obstructed labor^†^ | |  | |  | |  | |  | |  | |  | |  | |  | |  | |  | | |  | |  |
| Q215_4 | | Ruptured uterus | |  | |  | |  | |  | |  | |  | |  | |  | |  | |  | | |  | |  |
| Q216_4 | | Postpartum sepsis | |  | |  | |  | |  | |  | |  | |  | |  | |  | |  | | |  | |  |
| Q217_4 | | Severe pre-eclampsia/eclampsia | |  | |  | |  | |  | |  | |  | |  | |  | |  | |  | | |  | |  |
| Q218_4 | | Severe abortion complications (severe hemorrhage, infection/sepsis, uterine perforation, organ injury) | |  | |  | |  | |  | |  | |  | |  | |  | |  | |  | | |  | |  |
| Q219_4 | | Ectopic pregnancy | |  | |  | |  | |  | |  | |  | |  | |  | |  | |  | | |  | |  |
| Q220_4 | | Other direct obstetric complications[[1]](#footnote-2) | |  | |  | |  | |  | |  | |  | |  | |  | |  | |  | | |  | |  |
| **Indirect Obstetric Complications** | | | | | | | | | | | | | | | | | | | | | | | | | | | |
| Q221_4 | | Malaria | |  | |  | |  | |  | |  | |  | |  | |  | |  | |  | | |  | |  |
| Q222_4 | | HIV/AIDS-related | |  | |  | |  | |  | |  | |  | |  | |  | |  | |  | | |  | |  |
| Q223_4 | | Severe anemia | |  | |  | |  | |  | |  | |  | |  | |  | |  | |  | | |  | |  |
| Q224_4 | | Hepatitis | |  | |  | |  | |  | |  | |  | |  | |  | |  | |  | | |  | |  |
| Q225_4 | | Other indirect complications[[2]](#footnote-3)** | |  | |  | |  | |  | |  | |  | |  | |  | |  | |  | | |  | |  |
| **Maternal Deaths Due to Direct Obstetric Causes** | | | | | | | | | | | | | | | | | | | | | | | | | | | |
| Q226_4 | Antepartum hemorrhage | |  | |  | |  | |  | |  | |  | |  | |  | |  | |  | |  | | |  | |
| Q227_4 | Postpartum hemorrhage | |  | |  | |  | |  | |  | |  | |  | |  | |  | |  | |  | | |  | |
| Q228_4 | Retained placenta | |  | |  | |  | |  | |  | |  | |  | |  | |  | |  | |  | | |  | |
| Q229_4 | Obstructed/prolonged labor | |  | |  | |  | |  | |  | |  | |  | |  | |  | |  | |  | | |  | |
| Q230_4 | Ruptured uterus | |  | |  | |  | |  | |  | |  | |  | |  | |  | |  | |  | | |  | |
| Q231_4 | Post-partum sepsis | |  | |  | |  | |  | |  | |  | |  | |  | |  | |  | |  | | |  | |
| Q232_4 | Severe pre-eclampsia/eclampsia | |  | |  | |  | |  | |  | |  | |  | |  | |  | |  | |  | | |  | |
| Q233_4 | Abortion complications | |  | |  | |  | |  | |  | |  | |  | |  | |  | |  | |  | | |  | |
| Q234_4 | Ectopic pregnancy | |  | |  | |  | |  | |  | |  | |  | |  | |  | |  | |  | | |  | |
| Q235_4 | Other maternal deaths due to direct causes[[3]](#footnote-4)*** | |  | |  | |  | |  | |  | |  | |  | |  | |  | |  | |  | | |  | |
| **Maternal Deaths Due to Indirect Obstetric Causes** | | | | | | | | | | | | | | | | | | | | | | | | | | | |
| Q236_4 | Malaria | |  | |  | |  | |  | |  | |  | |  | |  | |  | |  | |  | | |  | |
| Q237_4 | HIV/AIDS-related | |  | |  | |  | |  | |  | |  | |  | |  | |  | |  | |  | | |  | |
| Q238_4 | Severe anemia | |  | |  | |  | |  | |  | |  | |  | |  | |  | |  | |  | | |  | |
| Q239_4 | Hepatitis | |  | |  | |  | |  | |  | |  | |  | |  | |  | |  | |  | | |  | |
| Q240_4 | Other indirect causes[[4]](#footnote-5)**** | |  | |  | |  | |  | |  | |  | |  | |  | |  | |  | | |  | |  | |
| Maternal Deaths Due to Unknown or Unspecified Causes | | | | | | | | | | | | | | | | | | | | | | | | | | | |
| Q241_4 | Unknown/unspecified causes | |  | |  | |  | |  | |  | |  | |  | |  | |  | |  | | |  | |  | |
| **Newborn Outcomes (for Facility Births)** | | | | | | | | | | | | | | | | | | | | | | | | | | | |
| Q242_4 | Live births ≥2500 grams | |  | |  | |  | |  | |  | |  | |  | |  | |  | |  | | |  | |  | |
| Q243_4 | Live births (2000 – 2499 grams) | |  | |  | |  | |  | |  | |  | |  | |  | |  | |  | | |  | |  | |
| Q244a_4 | Live births (1500-1999 grams) | |  | |  | |  | |  | |  | |  | |  | |  | |  | |  | | |  | |  | |
| Q244b_4 | Live births (<1500 grams) | |  | |  | |  | |  | |  | |  | |  | |  | |  | |  | | |  | |  | |
| Q245_4 | Live births, unspecified birth weight | |  | |  | |  | |  | |  | |  | |  | |  | |  | |  | | |  | |  | |
| Q246_4 | Preterm live births (<37 weeks) | |  | |  | |  | |  | |  | |  | |  | |  | |  | |  | | |  | |  | |
| Q247_4 | Stillbirths (intrapartum, macerated, unspecified) | |  | |  | |  | |  | |  | |  | |  | |  | |  | |  | | |  | |  | |
| **Neonatal Deaths** | | | | | | | | | | | | | | | | | | | | | | | | | | | |
| Q248_4 | Early neonatal deaths (1^st^ 24 hours; ≥2500 g) | |  | |  | |  | |  | |  | |  | |  | |  | |  | |  | | |  | |  | |
| Q249_4 | Early neonatal deaths (1^st^ 24 hours; 2000-2499 g) | |  | |  | |  | |  | |  | |  | |  | |  | |  | |  | | |  | |  | |
| Q250a_4 | Early neonatal deaths (1^st^ 24 hours; 1500-1999 g) | |  | |  | |  | |  | |  | |  | |  | |  | |  | |  | | |  | |  | |
| Q250b_4 | Early neonatal deaths (1^st^ 24 hours; <1500 g) | |  | |  | |  | |  | |  | |  | |  | |  | |  | |  | | |  | |  | |
| Q251_4 | Early neonatal deaths (1^st^ 24 hours; unspecified birth weight) | |  | |  | |  | |  | |  | |  | |  | |  | |  | |  | | |  | |  | |
| Q252_4 | Early neonatal deaths (1 to <7 days) | |  | |  | |  | |  | |  | |  | |  | |  | |  | |  | | |  | |  | |
| Q253_4 | Neonatal deaths (7 days to <28 days) | |  | |  | |  | |  | |  | |  | |  | |  | |  | |  | | |  | |  | |
| **Newborn Morbidities** | | | | | | | | | | | | | | | | | | | | | | | | | | | |
| Q254_4 | Newborns initiating kangaroo mother care (KMC) | |  | |  | |  | |  | |  | |  | |  | |  | |  | |  | | |  | |  | |
| Q255_4 | Newborns receiving resuscitation with bag & mask | |  | |  | |  | |  | |  | |  | |  | |  | |  | |  | | |  | |  | |
| **Referrals** | | | | | | | | | | | | | | | | | | | | | | | | | | | |
| Q256_4 | Emergency referrals **out** of this facility due to obstetric indications[[5]](#footnote-6)***** | |  | |  | |  | |  | |  | |  | |  | |  | |  | |  | | |  | |  | |
| Q257_4 | Emergency referrals **out** of this facility due to newborn indications***** | |  | |  | |  | |  | |  | |  | |  | |  | |  | |  | | |  | |  | |

SECTION 3: Quality of Registry Data

| **No.** | **Question** | **Are all columns completed?** | | | **Is register up-to-date?** | | |
| --- | --- | --- | --- | --- | --- | --- | --- |
|  | | **Yes** | **No** | **Not available** | **Yes** | **No** | **Not available** |
| 3.01 | Based on your observations, would you say that the **labor and** **delivery ward register:** | 1 | 0 | 9 | 1 | 0 | 9 |
| 3.02 | Based on your observations, would you say that the **safe abortion/postabortion register:** | 1 | 0 | 9 | 1 | 0 | 9 |
| 3.03 | Based on your observations, would you say that the **operating theater register:** | 1 | 0 | 9 | 1 | 0 | 9 |

| **Comments** |
| --- |
|  |

1. ^†^ Arrest disorder (arrest of cervical dilatation, arrest of descent), protraction disorder (protracted cervical dilatation, protracted descent), prolonged latent first stage, delayed/prolonged second stage, “CPD”, malpresentation such as transverse presentation

   *Examples of other direct complications include: premature rupture of membranes, preterm labor, post-term labor, previous cesarean, cord prolapse, breech, and multiple gestations. [↑](#footnote-ref-2)
2. ^**^ Examples of other indirect complications include: typhoid, cardiac disease, diabetes (including gestational diabetes), tuberculosis (TB), etc. [↑](#footnote-ref-3)
3. ^***^Examples of other maternal deaths due to direct causes include: embolism, anesthesia, suicide, etc. [↑](#footnote-ref-4)
4. ^****^ Examples of maternal death due to other indirect causes include: cardiac disease, diabetes (including gestational diabetes), TB, etc. [↑](#footnote-ref-5)
5. ^*****^ If a referral was made for both obstetric **and** newborn indications, count it under Row 68 (obstetric indications). [↑](#footnote-ref-6)
