# Supplementary material for: Cesarean delivery rates, hospital readiness and quality of clinical management in Ethiopia: national results from two cross-sectional emergency obstetric and newborn care assessments
Source: BMC Pregnancy Childbirth. 2021 Aug 19;21:571. doi: 10.1186/s12884-021-04008-9 (PMC8377989; doi:10.1186/s12884-021-04008-9)
Supplement: Supplementary file 5 — Additional file 5: Module 5. EmOC and EmNeC Signal Functions and Other Essential Services. [file 12884_2021_4008_MOESM5_ESM.doc]

EmONC Assessment

MODULE 5: EmOC and EmNeC Signal Functions and Other Essential Services

**Interviewer Name**

**Date** (dd/mm/yyyy): ___ / ___ / ___

**Instructions:** *Answer the following questions regarding the EmOC and EmNeC Signal Functions by interviewing health workers in the maternity ward and other departments, reviewing facility registers, and through observation. For many questions, you will record whether the function has been performed in the past 3 months, and if not, why it has not been performed.*[[1]](#footnote-2)* *Remember that “parenteral” means by injection, either intramuscular or intravenous.*

Section 1. Emergency Obstetric Signal Functions

| **No.** | **Item** | **Responses** | | **Skip to** |
| --- | --- | --- | --- | --- |
| **Parenteral antibiotics** | | | | |
| Q101_5 | Have parenteral antibiotics been administered to a pregnant or recently delivered woman in the last 3 months? | Yes 1  No 0 | | **If “Yes,” skip to Q103_5** |
| Q102_5 | If parenteral antibiotics were NOT administered in the last 3 months, why?  ***(circle 1 for all spontaneous*)** | Spontaneously mentioned | Not mentioned |  |
|  | a. lack of human resources | 1 | 0 |  |
|  | b. training needed | 1 | 0 |  |
|  | c. lack of supplies/equipment/drugs | 1 | 0 |  |
|  | d. weak management | 1 | 0 |  |
|  | e. unsupportive or no policy | 1 | 0 |  |
|  | f. no indication | 1 | 0 |  |
|  | g. other (specify) _____________ | 1 | 0 |  |

| **No.** | **Item** | **Responses** | | | | | **Skip to** |
| --- | --- | --- | --- | --- | --- | --- | --- |
| **Parenteral uterotonics** | | | | | | | |
| Q103_5 | Have parenteral uterotonics been administered in the last 3 months? | Yes 1  No 0 | | | | | **If “No,” skip to Q106_5** |
| Q104_5 | If parenteral uterotonics were administered in the last 3 months, which type of uterotonic was used?  ***(circle one)*** | Oxytocin 1  Ergometrine 2  Both 3  Other *(specify)* 4  ______________________________ | | | | |  |
| Q105_5 | Were they used … | Yes | No | | | | **All responses to this item skip to Q107_5** |
|  | 1. for labor induction/augmentation? (only oxytocin) | 1 | 0 | | | |  |
|  | 1. to prevent postpartum hemorrhage? | 1 | 0 | | | |  |
|  | 1. to treat postpartum hemorrhage? | 1 | 0 | | | |  |
| Q106_5 | If parenteral uterotonics were NOT administered in the last 3 months, why?  ***(circle 1 for all spontaneous answers; otherwise circle 0)*** | Spontaneously  Mentioned | | Not mentioned | | |  |
|  | a. lack of human resources | 1 | | 0 | | |  |
|  | b. training needed | 1 | | 0 | | |  |
|  | c. lack of supplies/equipment/drugs | 1 | | 0 | | |  |
|  | d. weak management | 1 | | 0 | | |  |
|  | e. unsupportive or no policy | 1 | | 0 | | |  |
|  | f. no indication | 1 | | 0 | | |  |
|  | g. other (specify)_________________ | 1 | | 0 | | |  |
| Q107_5 | Is misoprostol used in this facility for obstetric/gynecological indications? | Yes 1  No 0 | | | | | **If No, skip to Q109_5** |
| Q108_5 | Was it used … | Yes | No | | | |  |
|  | 1. For labor induction? | 1 | 0 | | | |  |
|  | 1. To prevent postpartum hemorrhage? | 1 | 0 | | | |  |
|  | 1. To treat postpartum hemorrhage? | 1 | 0 | | | |  |
|  | 1. To treat incomplete abortion? | 1 | 0 | | | |  |
|  | 1. To terminate pregnancy | 1 | 0 | | | |  |
| Q109_5 | Does staff routinely practice active management of the third stage of labor? | Yes 1  No 0 | | | | |  |
| **Parenteral anticonvulsants** | | | | | | | |
| Q110_5 | Have parenteral anticonvulsants been administered in the last 3 months? | Yes 1  No 0 | | | | | **If “No,” skip to Q113_5** |
| Q111_5 | If parenteral anticonvulsants were administered in the last 3 months, which type of anticonvulsant was used?  ***(circle one)*** | Magnesium sulfate 1  Diazepam 2  Both 3  Other *(specify)* 96  ______________________________ | | | | |  |
| Q112_5 | What health workers provided anticonvulsants in the last 3 months?  ***(circle 1 for all spontaneous answers; otherwise circle 0)*** | Spontaneously  Mentioned | | Not mentioned | | | **All responses to this item skip to Q114_5** |
|  | a. medical doctor (general practitioner) | 1 | | 0 | | |  |
|  | b. ob/gyn | 1 | | 0 | | |  |
|  | 1. health officer | 1 | | 0 | | |  |
|  | 1. emergency surgical officer | 1 | | 0 | | |  |
|  | 1. anesthetist | 1 | | 0 | | |  |
|  | 1. midwife | 1 | | 0 | | |  |
|  | 1. nurse | 1 | | 0 | | |  |
|  | 1. other (specify)__________________ | 1 | | 0 | | |  |
| Q113_5 | If parenteral anticonvulsants were NOT administered in the last 3 months, why?  ***(circle 1 for all spontaneous answers; otherwise circle 0)*** | Spontaneously  Mentioned | | Not mentioned | | |  |
|  | a. lack of human resources | 1 | | 0 | | |  |
|  | b. training needed | 1 | | 0 | | |  |
|  | c. lack of supplies/equipment/drugs | 1 | | 0 | | |  |
|  | d. weak management | 1 | | 0 | | |  |
|  | e. unsupportive or no policy | 1 | | 0 | | |  |
|  | f. no indication | 1 | | 0 | | |  |
|  | 1. other (specify)   ___________________________ | 1 | | 0 | | |  |
| **Manual removal of placenta** | | | | | | | |
| Q114_5 | Has manual removal of placenta been performed in the last 3 months? | Yes 1  No 0 | | | | | **If “No,” skip to Q116_5** |
| Q115_5 | What health workers provided manual removal of the placenta in the last 3 months?  ***(circle 1 for all spontaneous answers; otherwise circle 0)*** | Spontaneously  Mentioned | | | Not mentioned | | **All responses to this item skip to Q117_5** |
|  | a. medical doctor (general practitioner) | 1 | | | 0 | |  |
|  | b. ob/gyn | 1 | | | 0 | |  |
|  | 1. health officer | 1 | | | 0 | |  |
|  | 1. emergency surgical officer | 1 | | | 0 | |  |
|  | 1. anesthetist | 1 | | | 0 | |  |
|  | 1. midwife | 1 | | | 0 | |  |
|  | 1. nurse | 1 | | | 0 | |  |
|  | 1. other (specify)__________________ | 1 | | | 0 | |  |
| Q116_5 | If manual removal of placenta was NOT performed in the last 3 months, why?  ***(circle 1 for all spontaneous  answers; otherwise circle 0)*** | Spontaneously  Mentioned | | | Not mentioned | |  |
|  | a. lack of human resources | 1 | | | 0 | |  |
|  | b. training needed | 1 | | | 0 | |  |
|  | c. lack of supplies/equipment/drugs | 1 | | | 0 | |  |
|  | d. weak management | 1 | | | 0 | |  |
|  | e. unsupportive or no policy | 1 | | | 0 | |  |
|  | f. no indication | 1 | | | 0 | |  |
|  | g. other (specify)  ____________________________ | 1 | | | 0 | |  |
| **Removal of retained products** | | | | | | | |
| Q117_5 | Has removal of retained products been performed in the last 3 months? | Yes 1  No 0 | | | | | **If “No,” skip to Q120_5** |
| Q118_5 | If removal of retained products was performed in last 3 months, which method was used?  ***(read options)*** | Yes | | | | No |  |
|  | a. manual vacuum aspiration | 1 | | | | 0 |  |
|  | b. electric vacuum aspiration | 1 | | | | 0 |  |
|  | c. dilation and curettage (D&C) | 1 | | | | 0 |  |
|  | 1. evacuation and curettage (E&C) | 1 | | | | 0 |  |
|  | 1. misoprostol | 1 | | | | 0 |  |
|  | 1. oxytocin | 1 | | | | 0 |  |
| Q119_5 | What health workers provided removal of retained products in the last 3 months?  ***(circle 1 for all spontaneous answers; otherwise circle 0)*** | Spontaneously  Mentioned | | | | Not mentioned | **All responses to this item skip to Q121_5** |
|  | 1. medical doctor (general practitioner) | 1 | | | | 0 |  |
|  | 1. ob/gyn | 1 | | | | 0 |  |
|  | 1. health officer | 1 | | | | 0 |  |
|  | 1. emergency surgical officer | 1 | | | | 0 |  |
|  | 1. anesthetist | 1 | | | | 0 |  |
|  | 1. midwife | 1 | | | | 0 |  |
|  | 1. nurse | 1 | | | | 0 |  |
|  | 1. Other (specify)__________________ | 1 | | | | 0 |  |
| Q120_5 | If removal of retained products was NOT performed in the last 3 months, why?  ***(circle 1 for all spontaneous answers; otherwise circle 0)*** | Spontaneously  Mentioned | | | | Not mentioned |  |
|  | a. lack of human resources | 1 | | | | 0 |  |
|  | b. training needed | 1 | | | | 0 |  |
|  | c. lack of supplies/equipment/drugs | 1 | | | | 0 |  |
|  | d. weak management | 1 | | | | 0 |  |
|  | e. unsupportive or no policy | 1 | | | | 0 |  |
|  | 1. Lack of lighting | 1 | | | | 0 |  |
|  | 1. Lack of electricity | 1 | | | | 0 |  |
|  | 1. no indication | 1 | | | | 0 |  |
|  | 1. other (*specify*)_____________ | 1 | | | | 0 |  |
| **Assisted vaginal delivery** | | | | | | | |
| Q121_5 | Has assisted vaginal delivery (by vacuum extraction or forceps) been performed in the last 3 months? | Yes 1  No 0 | | | | | **If “No,” skip to Q124_5** |
| Q122_5 | If assisted vaginal delivery was performed in the last 3 months, what instrument was used?  ***(circle one)*** | Vacuum extractor 1  Forceps 2  Both 3 | | | | |  |
| Q123_5 | What health workers managed assisted vaginal delivery with vacuum extraction or forceps in the last 3 months?  ***(circle 1 for all spontaneous answers; otherwise circle 0)*** | Spontaneously  Mentioned | | Not mentioned | | | **All responses to this item skip to Q125_5** |
|  | 1. medical doctor (general practitioner) | 1 | | 0 | | |  |
|  | 1. ob/gyn | 1 | | 0 | | |  |
|  | 1. health officer | 1 | | 0 | | |  |
|  | 1. emergency surgical officer | 1 | | 0 | | |  |
|  | 1. midwife | 1 | | 0 | | |  |
|  | 1. nurse | 1 | | 0 | | |  |
|  | 1. other (*specify*)   _________________________ | 1 | | 0 | | |  |
| Q124_5 | If vacuum extraction or forceps delivery was NOT performed in the last 3 months, why?  ***(circle 1 for all spontaneous answers; otherwise circle 0)*** | Spontaneously  Mentioned | | Not mentioned | | |  |
|  | a. lack of human resources | 1 | | 0 | | |  |
|  | b. training needed | 1 | | 0 | | |  |
|  | c. lack of supplies/equipment/drugs | 1 | | 0 | | |  |
|  | d. weak management | 1 | | 0 | | |  |
|  | e. unsupportive or no policy | 1 | | 0 | | |  |
|  | 1. Lack of lighting | 1 | | 0 | | |  |
|  | 1. Lack of electricity | 1 | | 0 | | |  |
|  | 1. no indication | 1 | | 0 | | |  |
|  | 1. other (*specify*)   ___________________________ | 1 | | 0 | | |  |
| **Cesarean delivery** | | | | | | | |
| Q125_5 | Has a cesarean section been performed in the last 3 months? | Yes 1  No 0 | | | | | **If “No,” skip to  Q130_5** |
| Q126_5 | On average, how long would it take staff to start a cesarean section if you make the decision to start one right now?  ***(888 = does not know; 999 = at this moment in time they could not perform a cesarean****)* | |___ |___|___| minutes | | | | |  |
| Q127_5 | What type of anesthesia is currently used when performing a cesarean delivery?  ***(read options out loud)*** | Yes | | No | | |  |
|  | a. general | 1 | | 0 | | |  |
|  | b. spinal | 1 | | 0 | | |  |
|  | c. epidural | 1 | | 9 | | |  |
|  | d. ketamine | 1 | | 0 | | |  |
|  | e. other (*specify*) ___________________ | 1 | | 0 | | |  |
| Q128_5 | What health workers provided cesarean section in the last 3 months?  ***(circle 1 for all spontaneous answers; otherwise circle 0)*** | Spontaneously  Mentioned | | Not mentioned | | |  |
|  | a. medical doctor (general practitioner) | 1 | | 0 | | |  |
|  | b. ob/gyn | 1 | | 0 | | |  |
|  | c. general surgeon | 1 | | 0 | | |  |
|  | d. emergency surgical officer | 1 | | 0 | | |  |
|  | 1. health officer | 1 | | 0 | | |  |
|  | f. other (*specify*)______________ | 1 | | 0 | | |  |
| Q129_5 | What health workers provided anesthesia in the last 3 months?  ***(circle 1 for all spontaneous answers; otherwise circle 0)*** | Spontaneously  Mentioned | | Not mentioned | | | **All responses to this item skip to Q131_5** |
|  | a. medical doctor (general practitioner) | 1 | | 0 | | |  |
|  | b. ob/gyn | 1 | | 0 | | |  |
|  | c. emergency surgical officer | 1 | | 0 | | |  |
|  | 1. health officer | 1 | | 0 | | |  |
|  | e. anesthesiologist | 1 | | 0 | | |  |
|  | f. anesthetist | 1 | | 0 | | |  |
|  | g. other (*specify*)_______________ | 1 | | 0 | | |  |
| Q130_5 | If a cesarean was NOT performed in the last 3 months, why?  **(circle 1 for all spontaneous answers; otherwise circle 0)** | Spontaneously  Mentioned | | Not mentioned | | |  |
|  | a. lack of human resources | 1 | | 0 | | |  |
|  | b. training needed | 1 | | 0 | | |  |
|  | c. lack of supplies/equipment/drugs | 1 | | 0 | | |  |
|  | d. weak management | 1 | | 0 | | |  |
|  | e. unsupportive or no policy | 1 | | 0 | | |  |
|  | 1. Lack of lighting | 1 | | 0 | | |  |
|  | 1. Lack of electricity | 1 | | 0 | | |  |
|  | 1. no indication | 1 | | 0 | | |  |
|  | 1. other (*specify*)______________ | 1 | | 0 | | |  |
| **Blood transfusion** | | | | | | | |
| Q131_5 | Has blood transfusion been performed in the last 3 months? | Yes 1  No 0 | | | | | **If “No,” skip to Q134_5** |
| Q132_5 | If blood transfusion was performed in the last 3 months, describe the primary supply of blood.  ***(circle one)*** | Blood comes from central blood bank 1  Blood comes from a facility blood bank 2  Blood is collected from family or  friends as needed  (i.e., direct transfusion) 3  Other *(specify)* 96  ____________________________ | | | | |  |
| Q133_5 | What health workers provided blood transfusions in the last 3 months?  ***(circle 1 for all spontaneous answers; otherwise circle 0)*** | Spontaneously  Mentioned | | Not mentioned | | | **All responses to this item skip to Section 2** |
|  | a. medical doctor (general practitioner) | 1 | | 0 | | |  |
|  | b. ob/gyn | 1 | | 0 | | |  |
|  | c. emergency surgical officer | 1 | | 0 | | |  |
|  | d. health officer | 1 | | 0 | | |  |
|  | 1. midwife | 1 | | 0 | | |  |
|  | 1. nurse | 1 | | 0 | | |  |
|  | 1. laboratory technician | 1 | | 0 | | |  |
|  | 1. other (*specify*)   _______________________________ | 1 | | 0 | | |  |
| Q134_5 | If blood transfusion was NOT performed in the last 3 months, why?  ***(circle 1 for all spontaneous answers; otherwise circle 0)*** | Spontaneously  Mentioned | | Not mentioned | | |  |
|  | a. lack of human resources | 1 | | 0 | | |  |
|  | b. training needed | 1 | | 0 | | |  |
|  | c. lack of supplies/equipment/drugs | 1 | | 0 | | |  |
|  | d. weak management | 1 | | 0 | | |  |
|  | e. unsupportive or no policy | 1 | | 0 | | |  |
|  | 1. Lack of lighting | 1 | | 0 | | |  |
|  | 1. Lack of electricity | 1 | | 0 | | |  |
|  | h. no indication | 1 | | 0 | | |  |
|  | i. other (*specify*)_______________ | 1 | | 0 | | |  |

Section 2. Emergency Newborn Signal Functions and Other Newborn Interventions

| **No.** | | **Item** | | **Responses** | | | | **Skip to** |
| --- | --- | --- | --- | --- | --- | --- | --- | --- |
| **Newborn resuscitation** | | | | | | | | |
| Q201_5 | | Has newborn resuscitation with bag and mask been performed in the last 3 months? | | Yes 1  No 0 | | | | **If “No,” skip to Q 203_5** |
| Q202_5 | | What health workers provided neonatal resuscitation in the last 3 months?  *(****circle 1 for all spontaneous answers; otherwise circle 0****)* | | Spontaneously  Mentioned | | Not mentioned | | **All responses to this item skip to Q204_5** |
|  | | a. medical doctor (general practitioner) | | 1 | | 0 | |
|  | | b. pediatrician | | 1 | | 0 | |
|  | | c. neonatologist | | 1 | | 0 | |
|  | | d. ob/gyn | | 1 | | 0 | |
|  | | 1. anesthesiologist / anesthetist | | 1 | | 0 | |
|  | | 1. emergency surgical officer | | 1 | | 0 | |
|  | | 1. health officer | | 1 | | 0 | |
|  | | h. midwife | | 1 | | 0 | |
|  | | i.. nurse | | 1 | | 0 | |
|  | | i. other *(specify*)____________________ | | 1 | | 0 | |
| Q203_5 | | If newborn resuscitation with bag and mask was NOT performed in the last 3 months, why?  **(circle 1 for all spontaneous answers; otherwise circle 0**) | | Spontaneously  Mentioned | | Not mentioned | |  |
|  | | a. lack of human resources | | 1 | | 0 | |  |
|  | | b. training needed | | 1 | | 0 | |  |
|  | | c. lack of supplies/equipment/drugs | | 1 | | 0 | |  |
|  | | d. weak management | | 1 | | 0 | |  |
|  | | e. unsupportive or no policy | | 1 | | 0 | |  |
|  | | f. no indication | | 1 | | 0 | |  |
|  | | g. other *(specify)*  ___________________________ | | 1 | | 0 | |  |
| **Antenatal corticosteriods** | | | | | | | | |
| Q204_5 | Have antenatal corticosteroids been provided to manage pre-term labor in the last 3 months? | | Yes 1  No 0 | | | | **If “No,” skip to Q206_5** | |
| Q205_5 | What health workers provided corticosteroids in the last 3 months?  ***(circle 1 for all spontaneous answers; otherwise circle 0)*** | | Spontaneously  Mentioned | | Not mentioned | | **All responses to this item skip to Q207_5** | |
|  | a. medical doctor (general practitioner) | | 1 | | 0 | |
|  | b. pediatrician | | 1 | | 0 | |
|  | c. neonatologist | | 1 | | 0 | |
|  | d. ob/gyn | | 1 | | 0 | |
|  | e. emergency surgical officer | | 1 | | 0 | |
|  | f. health officer | | 1 | | 0 | |
|  | g. midwife | | 1 | | 0 | |
|  | h. nurse | | 1 | | 0 | |
|  | i. other *(specify)*  _________________________ | | 1 | | 0 | |
| Q206_5 | If corticosteroids for pre-term labor were NOT provided in the last 3 months, why?  ***(circle 1 for all spontaneous answers; otherwise circle 0)*** | | Spontaneously  Mentioned | | Not mentioned | |  | |
|  | a. lack of human resources | | 1 | | 0 | |  | |
|  | b. training needed | | 1 | | 0 | |  | |
|  | c. lack of supplies/equipment/drugs | | 1 | | 0 | |  | |
|  | d. weak management | | 1 | | 0 | |  | |
|  | e. unsupportive or no policy | | 1 | | 0 | |  | |
|  | f. no indication | | 1 | | 0 | |  | |
|  | g. other *(specify)*  ____________________________ | | 1 | | 0 | |  | |
| **Antibiotics for pPROM** | | | | | | | | |
| Q207_5 | Have antibiotics been given for preterm premature rupture of membranes (pPROM) in the last 3 months? | | Yes 1  No 0 | | | | **If “No,” skip to Q209_5** | |
| Q208_5 | What health workers provided antibiotics for pPROM in the last 3 months?  ***(circle 1 for all spontaneous answers; otherwise circle 0)*** | | Spontaneously  Mentioned | | Not mentioned | | **All responses to this item skip to Q210_5** | |
|  | a. medical doctor (general practitioner) | | 1 | | 0 | |
|  | b. pediatrician | | 1 | | 0 | |
|  | c. neonatologist | | 1 | | 0 | |
|  | d. ob/gyn | | 1 | | 0 | |
|  | e. emergency surgical officer  f. health officer | | 1 | | 0 | |
|  | g. midwife | | 1 | | 0 | |
|  | h. nurse | | 1 | | 0 | |
|  | i. other *(specify)*____________________ | | 1 | | 0 | |
| Q209_5 | If antibiotics for pPROM were NOT provided in the last 3 months, why?  ***(circle 1 for all spontaneous answers; otherwise circle 0****)* | | Spontaneously  Mentioned | | Not mentioned | |  | |
|  | a. lack of human resources | | 1 | | 0 | |  | |
|  | b. training needed | | 1 | | 0 | |  | |
|  | c. lack of supplies/equipment/drugs | | 1 | | 0 | |  | |
|  | d. weak management | | 1 | | 0 | |  | |
|  | e. unsupportive or no policy | | 1 | | 0 | |  | |
|  | f. no indication | | 1 | | 0 | |  | |
|  | g. other *(specify)___*_________________ | | 1 | | 0 | |  | |
| **Antibiotics for neonatal infections** | | | | | | | | |
| Q210_5 | Have antibiotics been given for neonatal infections in the last 3 months? | | Yes 1  No 0 | | | | **If “No,” skip to Q212_5** | |
| Q211_5 | What health workers provided antibiotics for neonatal infections in the last 3 months?  ***(circle 1 for all spontaneous answers; otherwise circle 0)*** | | Spontaneously  Mentioned | | Not mentioned | | **All responses to this item skip to Q213_5** | |
|  | a. medical doctor (general practitioner) | | 1 | | 0 | |
|  | b. pediatrician | | 1 | | 0 | |
|  | c. neonatologist | | 1 | | 0 | |
|  | d. ob/gyn | | 1 | | 0 | |
|  | e. emergency surgical officer  f. health officer | | 1 | | 0 | |
|  | g. midwife | | 1 | | 0 | |
|  | h. nurse | | 1 | | 0 | |
|  | i. other *(specify)* ____________________ | | 1 | | 0 | |
| Q212_5 | If antibiotics for neonatal infections were NOT given in the last 3 months, why?  ***(circle 1 for all spontaneous answers; otherwise circle 0)*** | | Spontaneously  Mentioned | | Not mentioned | |  | |
|  | a. lack of human resources | | 1 | | 0 | |  | |
|  | b. training needed | | 1 | | 0 | |  | |
|  | c. lack of supplies/equipment/drugs | | 1 | | 0 | |  | |
|  | d. weak management | | 1 | | 0 | |  | |
|  | e. unsupportive or no policy | | 1 | | 0 | |  | |
|  | f. no indication | | 1 | | 0 | |  | |
|  | 1. other (specify)   ____________________________ | | 1 | | 0 | |  | |
| **Kangaroo Mother Care** | | | | | | | | |
| Q213_5 | Has Kangaroo Mother Care (KMC) been provided to very small babies in the last 3 months, either continuous or intermittent KMC? | | Yes 1  No 0 | | | | **If “No,” skip to Q215_5** | |
| Q214_5 | What health workers helped provide KMC in the last 3 months?  ***(circle 1 for all spontaneous answers; otherwise circle 0)*** | | Spontaneously  Mentioned | | Not mentioned | | **All responses to this item skip to Q216_5** | |
|  | a. medical doctor (general practitioner) | | 1 | | 0 | |
|  | b. pediatrician | | 1 | | 0 | |
|  | c. neonatologist | | 1 | | 0 | |
|  | d. ob/gyn | | 1 | | 0 | |
|  | e. emergency surgical officer | | 1 | | 0 | |
|  | 1. health officer | | 1 | | 0 | |
|  | g. midwife | | 1 | | 0 | |
|  | h. nurse | | 1 | | 0 | |
|  | i. other *(specify)*  _________________________ | | 1 | | 0 | |
| Q215_5 | If KMC was NOT provided in the last 3 months, why?  *(****circle 1 for all spontaneous answers; otherwise circle 0)*** | | Spontaneously  Mentioned | | Not mentioned | |  | |
|  | a. lack of human resources | | 1 | | 0 | |  | |
|  | b. training needed | | 1 | | 0 | |  | |
|  | c. lack of supplies/equipment/drugs | | 1 | | 0 | |  | |
|  | d. weak management | | 1 | | 0 | |  | |
|  | e. unsupportive or no policy | | 1 | | 0 | |  | |
|  | f. no indication | | 1 | | 0 | |  | |
|  | g. other *(specify)*  ____________________________ | | 1 | | 0 | |  | |
| **Administration of oxygen** | | | | | | | | |
| Q216_5 | Has oxygen been administered to a newborn in the last 3 months? | | Yes 1  No 0 | | | | **If “No,” skip to Q219_5** | |
| Q217_5 | What health workers administered oxygen to a newborn in the last 3 months?  ***(circle 1 for all spontaneous answers; otherwise circle 0)*** | | Spontaneously  Mentioned | | Not mentioned | |  | |
|  | a. medical doctor (general practitioner) | | 1 | | 0 | |  | |
|  | b. pediatrician | | 1 | | 0 | |  | |
|  | c. neonatologist | | 1 | | 0 | |  | |
|  | d. Ob/gyn | | 1 | | 0 | |  | |
|  | e. emergency surgical officer | | 1 | | 0 | |  | |
|  | f. health officer | | 1 | | 0 | |  | |
|  | g. anesthetist | | 1 | | 0 | |  | |
|  | h. midwife | | 1 | | 0 | |  | |
|  | i. nurse | | 1 | | 0 | |  | |
|  | 1. other *(specify)*   ____________________________ | | 1 | | 0 | |  | |
| Q218_5 | Was oxygen therapy monitored by pulse oximetry? | | Yes 1  No 0  Uncertain 2 | | | | **All responses to this item skip to Q220_5** | |
| Q219_5 | If oxygen has NOT been given to a newborn in the last 3 months, why?  ***(circle 1 for all spontaneous answers; otherwise circle 0)*** | | Spontaneously  Mentioned | | Not mentioned | |  | |
|  | a. lack of human resources | | 1 | | 0 | |  | |
|  | b. training needed | | 1 | | 0 | |  | |
|  | c. lack of supplies/equipment/drugs | | 1 | | 0 | |  | |
|  | d. weak management | | 1 | | 0 | |  | |
|  | e. unsupportive or no policy | | 1 | | 0 | |  | |
|  | 1. Lack of lighting | | 1 | | 0 | |  | |
|  | 1. Lack of electricity | | 1 | | 0 | |  | |
|  | 1. no indication | | 1 | | 0 | |  | |
|  | i. other *(specify)*  ____________________________ | | 1 | | 0 | |  | |
| **Administration of IV fluids** | | | | | | | | |
| Q220_5 | Have IV fluids been administered to a newborn in the last 3 months? | | Yes 1  No 0 | | | | **If “No,” skip to Q222_5** | |
| Q221_5 | What health workers administered IV fluids to a newborn in the last 3 months?  ***(circle 1 for all spontaneous answers; otherwise circle 0)*** | | Spontaneously  Mentioned | | Not mentioned | | **All responses to this item skip to Q223_5** | |
|  | a. medical doctor (general practitioner) | | 1 | | 0 | |
|  | b. pediatrician | | 1 | | 0 | |
|  | c. neonatologist | | 1 | | 0 | |
|  | d. ob/gyn | | 1 | | 0 | |
|  | e. emergency surgical officer | | 1 | | 0 | |
|  | f. health officer | | 1 | | 0 | |
|  | g. anesthetist | | 1 | | 0 | |
|  | h. midwife | | 1 | | 0 | |
|  | i. nurse | | 1 | | 0 | |
|  | 1. other *(specify)*   ____________________________ | | 1 | | 0 | |
| Q222_5 | If IV fluids have NOT been given to a newborn in the last 3 months, why?  ***(circle 1 for all spontaneous answers; otherwise circle 0)*** | | Spontaneously  Mentioned | | Not mentioned | |  | |
|  | a. lack of human resources | | 1 | | 0 | |
|  | b. training needed | | 1 | | 0 | |
|  | c. lack of supplies/equipment/drugs | | 1 | | 0 | |
|  | d. weak management | | 1 | | 0 | |
|  | e. unsupportive or no policy | | 1 | | 0 | |
|  | f. no indication | | 1 | | 0 | |
|  | g. other *(specify)*  ____________________________ | | 1 | | 0 | |
| Q223_5 | Does staff routinely apply chlorhexidine gel to the newborn’s cord stump? | | Yes 1  No 0 | | | |  | |

Section 3. Other Maternal, Newborn and RH Practices and Services

*Instructions: Please answer the following questions about these other services. Record whether the function has been performed in the past 3 months and why not.*

| **No.** | **Item** | **Responses**  **Yes No** | | **Skip to** |
| --- | --- | --- | --- | --- |
| Q301_5 | Has alternative feeding (expressing breast milk and using a cup or spoon for feeding) been used for babies in the last 3 months? | 1 | 0 | **If “Yes,” skip to Q303_5** |
| Q302_5 | If not performed in last 3 months, why? |  |  |  |
|  | 1. lack of staff | 1 | 0 |  |
|  | b. training needed | 1 | 0 |  |
|  | c. lack of supplies / equipment issues | 1 | 0 |  |
|  | d. weak management | 1 | 0 |  |
|  | e. unsupportive or no policy | 1 | 0 |  |
|  | f. no indication/no clients | 1 | 0 |  |
| Q303_5 | Have ARVs been given to newborns in the maternity / labor ward (PMTCT) in the last 3 months? | 1 | 0 | **If “Yes,” skip to Q305_5** |
| Q304_5 | If not performed in last 3 months, why? |  |  |  |
|  | 1. lack of staff | 1 | 0 |  |
|  | b. training needed | 1 | 0 |  |
|  | c. lack of supplies / equipment issues | 1 | 0 |  |
|  | d. weak management | 1 | 0 |  |
|  | e. unsupportive or no policy | 1 | 0 |  |
|  | f. no indication/no clients | 1 | 0 |  |
| Q305_5 | Have ARVs been given to seropositive mothers in maternity / labor in the last 3 months? | 1 | 0 | **If “Yes,” skip to Q307_5** |
| Q306_5 | If not performed in last 3 months, why? |  |  |  |
|  | 1. lack of staff | 1 | 0 |
|  | b. training needed | 1 | 0 |
|  | c. lack of supplies / equipment issues | 1 | 0 |
|  | d. weak management | 1 | 0 |
|  | e. unsupportive or no policy | 1 | 0 |
|  | f. no indication/no clients | 1 | 0 |
| Q307_5 | Has a partograph been used to manage labor in the last 3 months? | 1 | 0 | **If “Yes,” skip to Q309_5** |
| Q308_5 | If not performed in last 3 months, why? |  |  |  |
|  | 1. lack of staff | 1 | 0 |  |
|  | b. training needed | 1 | 0 |  |
|  | c. lack of supplies / equipment issues | 1 | 0 |  |
|  | d. weak management | 1 | 0 |  |
|  | e. unsupportive or no policy | 1 | 0 |  |
|  | f. no indication/no clients | 1 | 0 |  |
| Q309_5 | Has a breech delivery been performed in the last 3 months? | 1 | 0 | **If “Yes,” skip to Q311_5** |
| Q310_5 | If not performed in last 3 months, why? |  |  |  |
|  | 1. lack of staff | 1 | 0 |  |
|  | b. training needed | 1 | 0 |  |
|  | c. lack of supplies / equipment issues | 1 | 0 |  |
|  | d. weak management | 1 | 0 |  |
|  | e. unsupportive or no policy | 1 | 0 |  |
|  | f. no indication/no clients | 1 | 0 |  |
| Q311_5 | Has an episiotomy been performed in the last 3 months? | 1 | 0 | **If “Yes,” skip to Q313_5** |
| Q312_5 | If not performed in last 3 months, why? |  |  |  |
|  | 1. lack of staff | 1 | 0 |  |
|  | b. training needed | 1 | 0 |  |
|  | c. lack of supplies / equipment issues | 1 | 0 |  |
|  | d. weak management | 1 | 0 |  |
|  | e. unsupportive or no policy | 1 | 0 |  |
|  | f. no indication/no clients | 1 | 0 |  |
| Q313_5 | Has a craniotomy or other form of destructive delivery been performed in the last 3 months? | 1 | 0 | **If “Yes,” skip to Q315_5** |
| Q314_5 | If not performed in last 3 months, why? |  |  |  |
|  | 1. lack of staff | 1 | 0 |  |
|  | b. training needed | 1 | 0 |  |
|  | c. lack of supplies / equipment issues | 1 | 0 |  |
|  | d. weak management | 1 | 0 |  |
|  | e. unsupportive or no policy | 1 | 0 |  |
|  | f. no indication/no clients | 1 | 0 |  |
| Q315_5 | Has a simple fistula been repaired in the last 3 months? | 1 | 0 | **If “Yes,” skip to Q317_5** |
| Q316_5 | If not performed in last 3 months, why? |  |  |  |
|  | 1. lack of staff | 1 | 0 |  |
|  | b. training needed | 1 | 0 |  |
|  | c. lack of supplies / equipment issues | 1 | 0 |  |
|  | d. weak management | 1 | 0 |  |
|  | e. unsupportive or no policy | 1 | 0 |  |
|  | f. no indication/no clients | 1 | 0 |  |
| Q317_5 | Have temporary family planning methods been provided in the last 3 months? | 1 | 0 | **If “Yes,” skip to Q319_5** |
| Q318_5 | If not performed in last 3 months, why? |  |  |  |
|  | 1. lack of staff | 1 | 0 |  |
|  | b. training needed | 1 | 0 |  |
|  | c. lack of supplies / equipment issues | 1 | 0 |  |
|  | d. weak management | 1 | 0 |  |
|  | e. unsupportive or no policy | 1 | 0 |  |
|  | f. no indication/no clients | 1 | 0 |  |
| Q319_5 | Have permanent contraception been provided in the last 3 months? | 1 | 0 | **If “Yes,” end the interview** |
| Q320_5 | If not performed in last 3 months, why? |  |  |  |
|  | 1. lack of staff | 1 | 0 |  |
|  | b. training needed | 1 | 0 |  |
|  | c. lack of supplies / equipment issues | 1 | 0 |  |
|  | d. weak management | 1 | 0 |  |
|  | e. unsupportive or no policy | 1 | 0 |  |
|  | f. no indication/no clients | 1 | 0 |  |

| **Comments:** |
| --- |
|  |

1. 1. ^^Lack of availability of necessary health workers
      - Required health workers are not posted to this facility in adequate numbers (or not at all)
   2. Training issues
      - Authorized cadre is available, but not trained
      - Providers lack confidence in their skills
   3. Supplies/equipment issues
      - Supplies/equipment are not available, not functional, or broken
      - Needed drugs are unavailable
   4. Management issues
      - Providers desire compensation to perform this function
      - Providers are encouraged to perform alternative procedures
      - Providers uncomfortable or unwilling to perform procedure for reasons unrelated to training
      - There is a lack of supervision
   5. Policy issues
      - National or facility policies do not allow function to be performed
   6. No indication
      - No client needing this procedure came to the facility during this time period

   [↑](#footnote-ref-2)
