# Supplementary material for: Cesarean delivery rates, hospital readiness and quality of clinical management in Ethiopia: national results from two cross-sectional emergency obstetric and newborn care assessments
Source: BMC Pregnancy Childbirth. 2021 Aug 19;21:571. doi: 10.1186/s12884-021-04008-9 (PMC8377989; doi:10.1186/s12884-021-04008-9)
Supplement: Supplementary file 6 — Additional file 6: Module 8. Cesarean Delivery Review. [file 12884_2021_4008_MOESM6_ESM.doc]

EmONC Assessment

MODULE 8: Cesarean Delivery Review

**Interviewer Name**

**Date** (dd/mm/yyyy): ___ / ___ / ___

**INSTRUCTIONS:** *Consult the birth register and/or the operating room log book to identify two cesarean deliveries for review. There is a column for each of the two women. Select the last two women who had a cesarean but who are no longer hospitalized among women who had cesareans in the last 12 months).*

*Put the response code for each question in the appropriate column. Ask to see the clinical card and partograph to verify each aspect assessed. If the response to the question is not documented in the labor and delivery register, operating theater log book, clinical record, or partograph, consider that the activity was not performed and use the code for “No/No information” (0 or 00) or the code. Otherwise, when information is missing, use the code for “No information (9 or 99). If cesareans are not performed at this facility, write “77” for each case in Row 1 and no cesarean review will be done. If only one case can be found, use “77” to show that the second case could not be found, and end the module.*

| **No.** | **Item** | **Case 1** | | **Case 2** | |
| --- | --- | --- | --- | --- | --- |
| Q101_8 | Age of the woman (77 = No case; 99 = No information) |  | |  | |
| Q102_8 | Parity of the woman (of index pregnancy)  1. Nulliparous (parity 0, this was her 1^st^ delivery)  2. Parity 1 (one previous delivery before this one)  3. Multiparous (2-4 previous deliveries)  4. Grand multiparous (> 5 previous deliveries)  9. No information |  | |  | |
| Q103_8 | Gestational age   1. Preterm (< 37 weeks) 2. Term (37-42 weeks) 3. Post term (>42 weeks) 4. No information |  | |  | |
| Q104_8 | Onset of labor  1. Spontaneous  2. Induced  3. Cesarean before labor  9. No information |  | |  | |
| Q105_8 | Cesarean was classified in the register/partograph/chart as:   1. Emergency 2. Elective 🡪 skip to Q107_8   9. No information 🡪 skip to Q107_8 |  | |  | |
| Q106_8 | How many hours and minutes elapsed between the decision to do an emergency cesarean and the beginning of surgery?  99 = No information | **l___l___l**  hours  **|___|___|**  minutes | | **|___|___|**  hours  **|___|___|**  minutes | |
| Q107_8 | Fetal presentation or lie  1. Cephalic  2. Breech  3. Transverse or oblique  9. No information |  | |  | |
| Q108_8 | Number of neonates   1. Singleton 2. Multiple 9. No information |  | |  | |
| Q109_8 | What was the primary indication for the cesarean?  1. Placenta previa  2. Placenta abruption  3. Arrest disorder (of cervical dilatation or of descent)  4. Failure to progress / protraction disorder  5. Prolonged latent first stage  6. Prolonged / delayed second stage  7. Cephalo-pelvic disproportion  8. Obstructed labor  9. Failed induction  10. Failed vacuum extraction or forceps  11. Failed trial of scar / failed VBACS  12. Previous CS / uterine scar  13. Uncontrolled severe pre-eclampsia / eclampsia  14. Cord prolapse  15. PMTCT of HIV  16. Fetal distress (persistent bradycardia, tachycardia, NRFHR)  17. Severe intrauterine growth restriction  18. Malpresentation, abnormal lie (transverse, oblique, brow, face)  19. Breech with footling  20. Other types of breech presentation  21. Multiple gestation  22. Vesico-vaginal fistula / fistula post repair  23. Meconium stained amniotic fluid in early stage of labor  24. Maternal medical disease  25. Maternal request  96. Other *(specify by writing in cell)*  99. No information |  | |  | |
| Q110_8 | How many previous cesarean deliveries did she have?  00 = No previous pregnancy; 99 = No information | |___|___| | | |___|___| | |
| Q111_8 | Was the woman referred from another health facility?  1. Yes 0. No / No information |  | |  | |
| Q112_8 | What was the HIV status of the woman?  1. Positive 2. Negative 3. Unknown |  | |  | |
| Q113_8 | Type of anesthesia used:  1. General  2. Spinal  3. Epidural  4. Ketamine only  9. No information |  | |  | |
| Q114_8 | What type of clinician provided the anesthesia?  1. Anesthesiologist  2. Anesthetist  3. Same person as did the surgery  96. Other *(specify by writing in cell)* |  | |  | |
| Q115_8 | What type of clinician performed the surgery?   1. Obstetrician/gynecologist 2. Emergency surgical officer 3. General practitioner 4. Health officer 5. General surgeon   9. No information  96. Other (*specify by writing in cell)* |  | |  | |
| Q116_8 | Was a partograph used to monitor labor (i.e., was a partograph filled out)?  0. Cesarean was elective, therefore no partograph  1. Yes, partograph used (type of partograph not important)  2. No, partograph not used  9. No information |  | |  | |
| Q117_8 | Was meconium present in the amniotic fluid?  1. Yes 0. No / No information |  | |  | |
| Q118_8 | What were the last three recorded fetal heart rates (beats per minute), ending with the last?  888 = Negative FHB (IUFD) 999 = No information | 1 __ __ __  2 __ __ __  3 __ __ __ | | 1 __ __ __  2 __ __ __  3 __ __ __ | |
| Q119_8 | What was the outcome for the newborn/newborns?  1. Normal live birth(s) 🡪 skip to Q122_8  2. Live birth with low Apgar score at 5^th^ min (< 6) 🡪 skip to Q122_8  3. Dead at birth (stillbirth) or died shortly afterward  4. One or more alive, one or more dead (twins or more)  9. No information 🡪 skip to Q122_8 |  | |  | |
| Q120_8 | Was the death a fresh stillbirth, macerated stillbirth or early neonatal death?  1. Fresh stillbirth(s)  2. Macerated stillbirth(s)  3. Very early neonatal death(s)  4. One or more stillbirths, one or more early neonatal deaths (twins or more)  9. No information 🡪 skip to Q122_8 |  | |  | |
| Q121_8 | If stillbirth/early neonatal death, what was the primary cause of death?  1. Preterm-related  2. Asphyxia  3. Infection/sepsis  4. Congenital anomalies  5. Birth trauma  8. Unknown  9. No information  96. Other *(specify by writing in cell)* |  | |  | |
| Q122_8 | What was the maternal outcome?  1. Alive 🡪 skip to Q125_8  2. Dead  9. No information 🡪 skip to Q125_8 |  | |  | |
| Q123_8 | If maternal death, what was the primary cause of death?  (please copy in the response column the number next to the primary cause of death as recorded in the patient card or other source of information)   1. APH 2. PPH 3. Retained placenta 4. Obstructed/prolonged labor 5. Ruptured uterus 6. Post-partum sepsis 7. Eclampsia 8. Severe complications of abortion 9. Ectopic pregnancy 10. Other direct complication (indicate code of response and specify other complication in cell) 11. Malaria 12. HIV/AIDS-related 13. Severe anemia 14. Hepatitis 15. Other indirect (indicate code of response and specify other complication in cell)   88. Cause of death unknown  99. No information on cause of death |  | |  | |
| Q124_8 | If maternal death, and there was another complication indicated as contributing to the death, what was the second contributing cause?  (please copy in the response column the number next to the secondary cause of death as recorded in the patient card or other source of information)   1. APH 2. PPH 3. Retained placenta 4. Obstructed/prolonged labor 5. Ruptured uterus 6. Post-partum sepsis 7. Eclampsia 8. Severe complications of abortion 9. Ectopic pregnancy 10. *Other direct complication* (indicate code of response and specify other complication in cell) 11. Malaria 12. HIV/AIDS-related 13. Severe anemia 14. Hepatitis 15. Other indirect (indicate code of response and specify other complication in cell)   99. No information on secondary cause of death |  | |  | |
| Q125_8 | Did the woman experience a complication during the operation?  1. Yes 0. No / No information 🡪 skip to Q127_8 |  | |  | |
| Q126_8 | What complications were there during the operation? | Yes | No | Yes | No |
|  | a. High spinal block | 1 | 0 | 1 | 0 |
|  | b. Problems with intubation | 1 | 0 | 1 | 0 |
|  | c. PPH | 1 | 0 | 1 | 0 |
|  | d. Bowel injury | 1 | 0 | 1 | 0 |
|  | e. Bladder injury | 1 | 0 | 1 | 0 |
|  | f. Ureter injury | 1 | 0 | 1 | 0 |
|  | 96. Other *(specify in cell*) |  | |  | |
| Q127_8 | Did the woman experience a complication after the operation?  1. Yes 0. No / No information 🡪 skip to Q129_8 |  | |  | |
| Q128_8 | What complications were there after the operation? | Yes | No | Yes | No |
|  | a. Wound infection | 1 | 0 | 1 | 0 |
|  | b Peritonitis/pelvic abscess | 1 | 0 | 1 | 0 |
|  | c. Re-laparotomy | 1 | 0 | 1 | 0 |
|  | 96. Other (*specify in cell*) |  | |  | |
| Q129_8 | Were prophylactic antibiotics administered before the C/S?  1. Yes 0. No / No information |  | |  | |
| Q130_8 | Were antibiotics administered after the C/S?  1. Yes 0. No / No information |  | |  | |
| Q131_8 | Were prophylactic uterotonics administered after the baby was delivered?  1. Yes 0. No / No information |  | |  | |
| Q132_8 | Anesthesia time in minutes  999 = No information | |___|___|___| | | |___|___|___| | |
|  |  |  | |  | |
| Q133_8 | Note the availability of documents used for this review | Yes | No | Yes | No |
|  | a. Preoperative decision note | 1 | 0 | 1 | 0 |
|  | b. Operation note | 1 | 0 | 1 | 0 |
|  | c. Anesthesia sheet | 1 | 0 | 1 | 0 |
|  | d. Recovery follow up sheet | 1 | 0 | 1 | 0 |
|  | e. Progress note | 1 | 0 | 1 | 0 |
|  | f. Discharge report/death summary | 1 | 0 | 1 | 0 |
|  | g. Safe surgical check list | 1 | 0 | 1 | 0 |
| Q134_8 | How long was the woman hospitalized before she was discharged (or died)?  *(write the number of days and hours)*  99 = No information | |____|____|  days  |____|____|  hours | | |____|____|  days  |____|____|  hours | |

| C**omments** |
| --- |
|  |
